# *Peperomia campylotropa* A.W. Hill: Ethnobotanical, Phytochemical, and Metabolomic Profile Related to Its Gastroprotective Activity

**DOI:** 10.3390/molecules30040772

**Published:** 2025-02-07

**Authors:** Yazmín K. Márquez-Flores, Jesús Ayala-Velasco, José Correa-Basurto, Alan Estrada-Pérez, M. Estela Meléndez-Camargo

**Affiliations:** 1 Laboratorio de Toxicología de Productos Naturales, Departamento de Farmacia, Escuela Nacional de Ciencias Biológicas, Campus Zacatenco, Instituto Politécnico Nacional, Av. Wilfrido Massieu s/n Col. Zacatenco, Ciudad de México C.P. 07738, Mexico; 2 Laboratorio de Farmacología Renal y Hepática, Departamento de Farmacia, Escuela Nacional de Ciencias Biológicas, Campus Zacatenco, Instituto Politécnico Nacional, Av. Wilfrido Massieu s/n, Col. Zacatenco, Ciudad de México C.P. 07730, Mexico; 3 Laboratorio de Diseño y Desarrollo de Nuevos Fármacos e Innovación Biotecnológica, Escuela Superior de Medicina, Instituto Politécnico Nacional, Plan de San Luis y Díaz Mirón s/n, Col. Casco de Santo Tomas, Ciudad de México C.P. 11340, Mexico; jcorreab@ipn.mx (J.C.-B.);

**Keywords:** *Peperomia campylotropa*, Piperaceae, gastroprotective, gastric ulcer

## Abstract

*Peperomia campylotropa* (Piperaceae) is a species with a traditional Mexican gastroprotective use that has never-before been studied using metabolomics. This study explores the ethnobotanical use of the species, aiming to define the gastroprotective effect of the aqueous extract and characterize its secondary metabolites by UHPLC–MS analysis. To validate its use, we botanically identified the species re-collected in the Municipality of Buenavista de Cuéllar, Guerrero, Mexico. We conducted interviews to provide evidence of the traditional details of its consumption and knowledge. Subsequently, qualitative phytochemical tests were performed to elucidate the possible secondary metabolites, which were also characterized under UHPLC–MS analysis and analyzed according to their primary type and retention times. Indomethacin (IND)- and ethanol (EtOH)-induced gastric damage models in Wistar rats were used for pharmacological evaluation, considering the ulceration index and gastroprotection percentage. Along with the participation in the mechanism of action of nitric oxide (NO), sulfhydryl (-SH) groups and prostaglandins (PG) were elucidated by Wistar rats pretreated with N(ω)-nitro-L-arginine methyl ester (L-NAME), N-Ethylmaleimide (NEM), and IND, respectively. Acute intragastric toxicity was also estimated in NIH female mice. Ninety people were interviewed, revealing the traditional knowledge of *P. campylotropa* as food and medicine for stomach diseases, including irritation and indigestion. The presence of phenolic compounds (48%), N-containing compounds (22%), glycosides (21%), terpenoids (7%), and lactones (4%) were verified by preliminary phytochemical analysis and by UHPLC–MS in which 162 secondary metabolites were characterized. Besides that, the aqueous extract at 62.5, 125, and 250 mg/kg of body weight (b.w.) decreased the ulcerative index, showing gastroprotection percentages between 60 and 80%, similar to that of omeprazole. Furthermore, -SH group participation in its activity was established. All this evidence supports the gastroprotective activity of *P. campylotropa* for the first time and contributes to understanding its secondary metabolite content.

## 1. Introduction

The Piperaceae family is widely distributed worldwide, mainly from Mexico to southwest Argentina [1]; however, several species, such as *Peperomia dindygulensis* Miq., *Piper guineense* (West African Black Pepper), and *Peperomia pellucida*, are distributed in tropical and subtropical regions of Asia, Africa, and Australia [2,3,4]. The *Peperomia* (1700 species) and *Piper* (2000 species) are the most representative; however, this family also includes the *Manekiam*, *Zippelia*, and *Verhuellia* genera [1].

According to Vergara-Rodríguez et al. (2017), this genus is not well-known in Mexico because of the many names attributed to it, even the use of illegitimate names, since the species is difficult to differentiate; so, the authors have reported 131 Mexican species, including those identified in Veracruz, Oaxaca, and Chiapas, the states with the highest diversity [5]. Traditionally, these species are used to treat inflammation, gastric ulcers, asthma, pain, gynecological illnesses, and intestinal disorders [1,6]; however, only a few studies corroborate some species’ effectiveness.

Various traditional Mexican pharmacological uses have been attributed to *Peperomia campylotropa* A.W. Hill, including as an analgesic agent, as an antispasmodic agent, and as a gastroprotective agent, an effect that has been demonstrated for other *Peperomia* species [2,7]; however, we are unaware of any studies showing the activity of this species.

Gastric ulcers are considered to be a global health problem, with an estimated prevalence of 5 to 10% of patients, a value that increases with age and is caused by several factors such as stress, smoking, nutritional deficiencies, *Helicobacter pylori* infection, and the consumption of alcohol or nonsteroidal anti-inflammatory drugs (NSAIDs) [8,9,10].

NSAIDs are one of the most common therapeutic groups used worldwide, although their use is associated with several digestive effects, including gastric mucosal erosions, ulcers, bleeding, and perforation. Their pathophysiology is related to the inhibition of the cyclooxygenase (COX) and the subsequent decrease in cytoprotective prostaglandin (PG) production [11].

Otherwise, it is well known that EtOH-induced gastric damage alters protective factors, such as through decreased mucus production and blood circulation within the mucosa, the generation of reactive species, and an exacerbated inflammatory response [12], consequently releasing endogenous mediators such as nitric oxide (NO), sulfhydryl groups (-SH), and PG.

Based on this, and considering that there is no evidence to prove the gastroprotective effect of *P. campylotropa*, we proposed evaluating their activity under chemically induced gastric damage in a rat model. Also, we emphasized the role of critical inflammatory endogenous mediators such as NO, -SH, and PG in terms of their mechanisms of action. In addition, to support their traditional uses, we performed an ethnobotanical study and a UHPLC–MS metabolomic analysis to contribute to the knowledge of the species’ secondary metabolite content.

## 2. Results

### 2.1. Ethnobotanical Study

**Figure 1:**
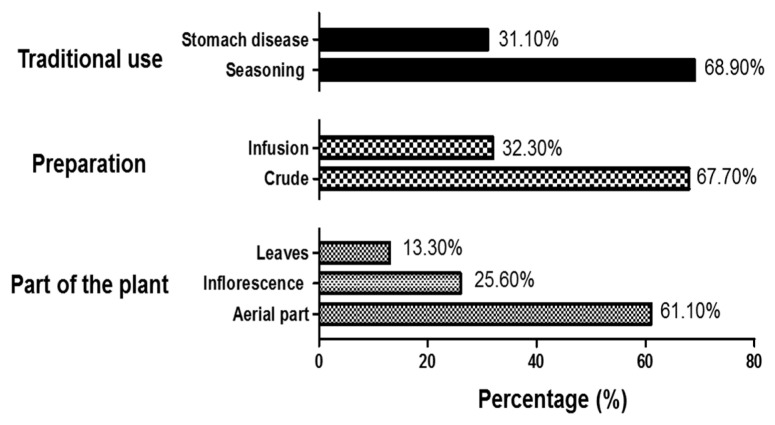
Traditional use of *P. campylotropa* in the Municipality of Buenavista de Cuéllar, Guerrero, Mexico; n = 90 people.

We interviewed 90 people who lived in the Municipality of Buenavista de Cuéllar, Guerrero, Mexico; of these, 93.33% (n = 84) corresponded to plant users, and 6.67% (n = 6) to plant sellers. The population knows the species as “cilantro de campo” and preferably uses the complete aerial part (61.11%), considering the leaves and inflorescence (spikes) (Figure 1).

As is shown in Figure 1, the species is commonly used in its crude form (67.70%) and as a seasoning (68.90%); however, 32.23% of people used it as an infusion. Regarding its pharmacological uses, 31.10% mentioned it was used for stomach diseases, including irritation and indigestion.

The amount used to treat an upset stomach is three to four leaves or large spikes in the form of an infusion, with a standard single-dose protocol being reported by 52.22% of the surveyed people: once a day for two or three days (36.66%) or during the period of discomfort (11.12%).

### 2.2. Preliminary Phytochemical Screening

The phytochemical screening conducted on *P. campylotropous* revealed the metabolites listed in Table 1.

### 2.3. Metabolomic Profile and Gastroprotective Metabolites of P. campylotropa

According to the UHPLC–MS analysis, 162 secondary metabolites were identified in *P. campylotropa*. The Supplementary Materials (Table S1) summarize the chemical information obtained. ordered by increasing retention time.

**Figure 2:**
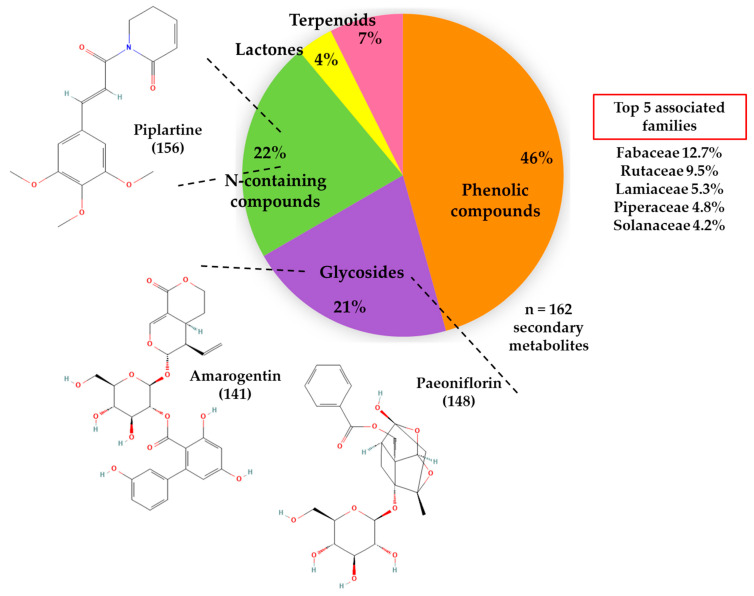
The primary type of secondary metabolites of aqueous extract of *P. campylotropa*. The graph represents the percentage of secondary metabolites grouped by type. Chemical structures obtained in PubChem emphasized three metabolites characterized in the extract, with previous antecedents of gastroprotective activity (Table S2); (number) = assigned number to the molecule considering its retention time (Table S1). Families correspond with those that were more commonly mentioned according to species related to the metabolites found (Table S2).

Otherwise, in Figure 2, we show the secondary metabolites grouped by primary type and emphasize three molecules mentioned previously in the literature for their gastroprotective activities: amarogentin, paeoniflorin, and piplartine. This species belongs to three families, namely Gentianaceae, Paeoniaceae, and Piperaceae, of the 55 families associated with the secondary metabolites characterized for *P. campylotropa* (Table S2). The most commonly mentioned families were Fabaceae, Rutaceae, Lamiaceae, Piperaceae, and Solanaceae. It is important to highlight that (E,E)-piperlonguminine (**37**), 3,4,5-trimethoxycinnamic acid (**80**), piperolactam D (**84**), piplartine (**156**), and pipercitine (**161**) are common metabolites of other Piperaceae family members.

### 2.4. Aqueous Extract of P. campylotropa Decreases Indomethacin-Induced Gastric Damage

**Figure 3:**
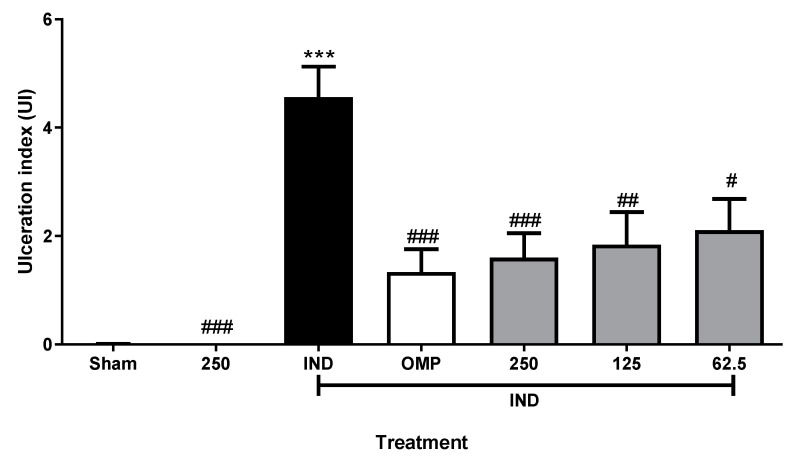
Effect of aqueous extract of *P. campylotropa* on ulceration index (UI) in IND-induced gastric damage model. Data are reported as means ± SEM for 6 animals. *** *p* < 0.001 = significantly different from the Sham group. ^#^ *p* < 0.05; ^##^ *p* < 0.01; and ^###^ *p* < 0.001 = significantly different from the IND group. One-way analysis of variance and Tukey’s Multiple Comparison Test were carried out. IND = 30 mg/kg of b.w.; OMP = 20 mg/kg of b.w.; gray bars represent groups treated with doses of 62.5, 125, and 250 mg/kg of b.w. of aqueous extract, respectively.

As is shown in Figure 3, IND (30 mg/kg of b.w.) increased the ulceration index (UI) (*p* < 0.001), an effect downregulated by OMP at a dosage of 20 mg/kg of b.w. A similar effect was observed in the *P. campylotropa* groups, highlighting that an extract of 250 mg/kg of b.w. did not induce ulcerogenic damage per se.

**Figure 4:**
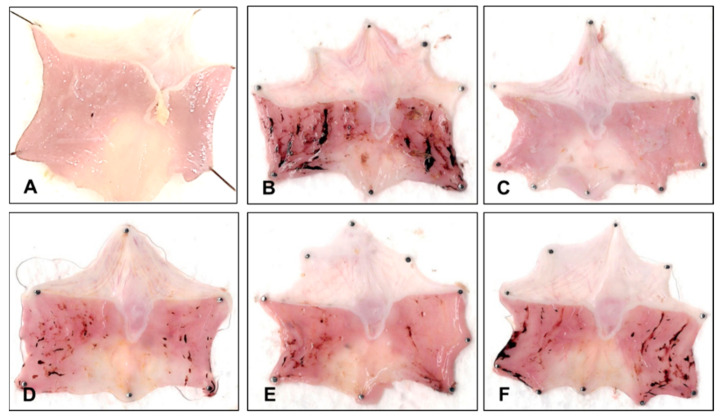
Effect of the aqueous extract of *P. campylotropa* on the macroscopic appearance of the gastric mucosa in rats after IND induction of gastric lesions. (**A**) Sham group; (**B**) IND 30 mg/kg of b.w.; (**C**) OMP 20 mg/kg of b.w.; (**D**–**F**) IND + *P. campylotropa* (250, 125, and 62.5 mg/kg of b.w., respectively).

Similar results were obtained considering the gastroprotection percentage (%) (Table 2), in which no differences were observed between the reference drug and the aqueous extract. Even the 250 mg/kg of b.w. doses of the aqueous extract showed a similar value (77.84 ± 12.20) compared to OMP (73.90 ± 11.11). Representative macroscopic images are shown in Figure 4, evidencing that macroscopic damage decreased with OMP and aqueous extract treatments.

### 2.5. Effect of Aqueous Extract of P. campylotropa on Absolute-EtOH-Induced Gastric Damage

**Figure 5:**
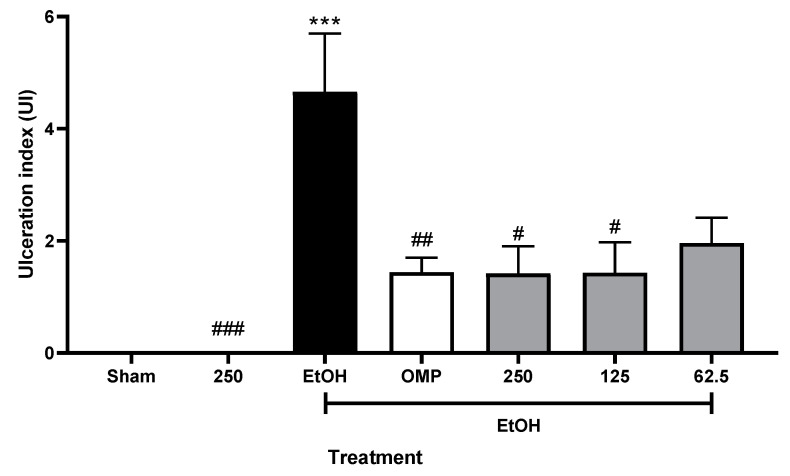
Effect of aqueous extract of *P. campylotropa* on ulceration index (UI) in EtOH-induced gastric damage model. Data are reported as means ± SEM for 6 animals. *** *p* < 0.001 = significantly different from the Sham group. ^#^ *p* < 0.05; ^##^ *p* < 0.01; and ^###^ *p* < 0.001 = significantly different from the EtOH group. One-way analysis of variance and Tukey’s Multiple Comparison Test were carried out. EtOH = 1 mL/250 g of b.w.; OMP = 20 mg/kg of b.w.; gray bars represent groups treated with doses (mg/kg of b.w.) of 62.5, 125, and 250 of aqueous extract, respectively.

Absolute EtOH can induce gastric damage by altering protective factors. As is shown in Figure 5, the intragastric administration of EtOH (1 mL/250 g of b.w.) significantly increased the ulceration index (*p* < 0.001). This effect was reversed by OMP (*p* < 0.01) and by treatment with 125 and 250 g/kg of the aqueous extract (*p* < 0.05).

Regarding the gastroprotection percentage (Table 2), *P. campylotropa* showed values higher than 60%, demonstrating the species’ gastroprotective activity.

### 2.6. Effect of L-NAME, NEM, and IND on the Gastroprotective Effect of P. campylotropa Aqueous Extract

Table 3 shows the effect of the pretreatment with L-NAME, NEM, and IND before EtOH induced gastric lesions. According to the expected, *P. campylotropa* (125 mg/kg of b.w.) significantly protected against 88.5% of the ethanol-induced gastric damage in rats without pretreatment with an inhibitor (NS-injected rats). Regarding the inhibitors, IND and L-NAME pretreatments did not modify the *P. campylotropa* gastroprotective activity; however, when we administered NEM as a pretreatment, the species completely lost its activity, demonstrating the participation of -SH groups in the gastroprotective mechanism of action of the aqueous extract.

### 2.7. Acute Intragastric Toxicity in Mice

No animal showed signs of toxicity or death, considering a possible acute intragastric toxicity (LD_50_) higher than 20 g/kg of b.w.

## 3. Discussion

Peperomia is a genus belonging to the Piperaceae family, which includes approximately 1700 identified species. In Mexico, 131 species have been documented; however, knowledge about them is limited due to their similarities and the difficulty in distinguishing between them [5]; among them, *P. campylotropa* A.W. Hill is a species that has been poorly studied. Our data represent, for the first time, experimental evidence that the gastroprotective effect of *P. campylotropa* A.W. Hill prevented IND- and EtOH-induced gastric mucosal injury, a therapeutic effect that has been attributed traditionally; in addition, its metabolomic profile revealed secondary molecules related to gastroprotective activity.

Firstly, to get solid and scientific evidence regarding the possible application of *P. campylotropa* as a gastroprotective agent, we performed ethnobotanical surveys of the population from Buenavista de Cuéllar, Guerrero, Mexico, to obtain traditional information about the species. Ethnobotany is “an interaction between people and plants in a given environment.” Based on this, ethnobotanical studies are relevant since they represent an approach to information from the past that enables the development of new drugs [14].

We reported that *P. campylotropa* is known as “cilantro de campo” and its whole aerial parts are used in crude form as a seasoning. However, it is also used by the population as an infusion for treating stomach disorders, including irritation and indigestion. This information is in accordance with previous reports in which, according to the uses of the Piperaceae family in Oaxaca, Mexico, the genus *Peperomia* is consumed fresh, as a food seasoning, and for medicinal treatments such as for acne elimination, childbirth recovery, toothache relief, stomach pain, and erysipelas symptoms, among others. Specifically, the authors reported the uses of *P. campylotropa* as being medicinal by the Cuicatec ethnic group [15].

Similarly, *P. pellucida* is consumed as a food and is considered to be a medicinal herb, used to treat gastric ulcers, gout, arthritis, and other ailments. Interestingly, its pharmacological effect has been corroborated by an EtOH-induced gastric-ulcer experimental rat model, identifying dichloromethane as the most potent extract and dillapiole as the most active compound [2].

We performed a preliminary phytochemical and metabolomic analysis of the *P. campylotropa* aqueous extract, revealing the presence of flavonoids, alkaloids, reducing sugars, tannins, and saponins. Except for the saponins, these compounds were also identified by UHPLC–MS analysis, in which the MS/MS spectrum chemically characterized 162 secondary metabolites. In this regard, phenolic compounds (46%), N-containing compounds (22%), glycosides (21%), terpenoids (7%), and lactones (4%) were the types of metabolites that were characterized.

As expected, several molecules have been reported within the Piperaceae family, mainly those related to the genus *Piper*, with which the genus *Peperomia* has a phylogenetic relationship with [16]. *Piper crassinervium*, *P. longum*, *P. retrofractum*, *P. khasianum*, *P. swartzianum*, *P. tuberculatum*, *P. arborescent*, *P. pubertum*, *P. nigrum*, *P. betle*, and *P. attenuatum* were the associated species. Interestingly, the last two have been reported for their gastroprotective properties.

The hydroalcoholic extract of *Piper betle* leaf was demonstrated using pyloric ligation and stress-induced antiulcer models in Wistar rats, with a similar effect to that of ranitidine [17]. Also, the hot aqueous extract (HAE) and cold ethanolic extract (CEE) of leaves grown in Sri Lanka show intense gastroprotective activity in an EtOH-induced gastric ulcer model in rats [7].

Similarly, Soni et al., 2023, reported the gastroprotective effect of the ethanolic leaf extract of *Piper attenuatum* in aspirin-induced gastric ulcers, emphasizing the presence of the phytoconstituents cepharadione A, cepharadione B, guineensine, norcepharadione B, and piperlonguminine [18]. In correlation with this species, we identified piperolactam D (**84**) as a metabolite of *P. campylotropa*, a similar alkaloid to piperolactam A identified in *P. attenuatum,* which showed a −8.3 kcal/mol binding affinity to the pig gastric H^+^/K^+^ ATPase protein (2XZB) compared to omeprazole (−7.3 kcal/mol) [18].

According to our data, *P. campylotropa* induced gastroprotection in both indomethacin- and ethanol-induced gastric ulcer models, with similar percentages to those of omeprazole (20 mg/kg of b.w.), a behavior that increased in concordance with increased doses (62.5, 125, 250 mg/kg of b.w.). Based on the ulceration index (UI), the *P. campylotropa* extract was most potent in the IND compared to the EtOH model.

Similar results were obtained with the aqueous suspension of the EtOH extract of the leaves of *Piper carpunya*, with the maximum effect at doses of 250 mg/kg of b.w. before diclofenac-induced gastric ulceration [19]; or for *P. betle* extracts with a significant decrease in length and number of gastric lesions induced by ethanol at doses of 200, 300, and 500 mg/kg of b.w. [7].

Several mechanisms of action have been described for gastroprotective drugs; among them are the reduction in gastric acid secretion by H_2_-receptor antagonists, the inhibition of the proton pump by proton pump inhibitors such as omeprazole, and the antibiotic effect against *H. pylori* [20].

Considering that gastric ulcers are lesions induced by an imbalance between aggressive and protective agents, and among the latter are the production of mucus, bicarbonate, and prostaglandins, separate experiments were performed to establish the role of endogenous nitric oxide (NO), sulfhydryl groups (-SH), and prostaglandins (PG) by oral pretreatment with L-NAME (a nitric oxide synthase inhibitor), NEM (a sulfhydryl compound blocker), and IND (a prostaglandin synthesis inhibitor), respectively.

EtOH administration provokes gastric necrotic damage and inflammation characterized by cell infiltration and reduction of the secretion of protective molecules, such as NO [21]. This is a crucial molecule due to several functions including the relaxation of the blood vessels on smooth muscle, an event that is involved in the maintenance of gastrointestinal mucosal integrity by regulating gastric mucosal blood flow, mucous secretion, and the defense barrier [22].

It has been reported that one of the gastroprotective mechanisms of medicinal plants is related to enhancing the production of NO to prevent the damage induced by necrotizing agents like ethanol or nonsteroidal anti-inflammatory drugs (NSAIDs) [23].

For this reason, we evaluated the role of endogenous NO in terms of the pharmacological effect of *P. campylotropa* by pretreatment with L-NAME, a nonspecific NOS (nitric oxide synthase) inhibitor. As was observed, the administration of L-NAME increased the gastric lesions induced by EtOH by 2-fold, an effect that was reversed by the administration of the aqueous extract (125 mg/kg; 57% gastroprotection); however, this gastroprotective percentage was lowest compared to the treatment alone (88.5% gastroprotection). These data demonstrated that NO is possibly involved in the mechanism of action of *P. campylotropa*.

Similarly, we used a NEM pretreatment to establish the role of the non-protein sulfhydryl groups (-SH). In this respect, NEM provoked an increase in ethanol-induced gastric damage; interestingly, the gastroprotective effect of *P. campylotropa* was abolished, which indicates the importance of -SH groups in their mechanism of action. -SH groups play an essential role in the stability of the gastric mucus since the disulfide bridges avoid mucus solubility and maintain the integrity of the mucus layer. An increase in disulfide-bridge damage is related to a consequent increase in susceptibility to damage [24].

Da Silva et al. (2016) mentioned that the *Piper umbellatum* L. gastroprotective effect is at least in part due to its antioxidant activity, acting on GSH (L-γ-glutamyl-L-cysteinyl glycine) levels, an endogenous sulfhydryl compound involved in the mucosal protection by controlling the composition of gastric mucus [25]. According to this, it will be essential to evaluate the antioxidant activity of *P. campylotropa* in further studies.

In another way, endogenous PG synthesized by COX, mainly the COX-1 isoform, is essential for the integrity of the gastric mucosa by the regulation of acid and bicarbonate secretion, mucus production, and mucosal blood flow [26]. In our case, the indomethacin pretreatment slightly increased the ulcerative index compared to the ethanol control group. This effect was decreased by the *P. campylotropa* oral treatment; however, the activity of the species was lowest compared to the group without pretreatment, an event that evidenced the participation of PG in the mechanism of action of the species.

Further, it has been described that the inhibition of PG synthesis disrupts the gastric mucosal blood flow, provoking a consequent up-regulation of the production of inflammatory mediators, among them are tumor necrosis factor-alpha (TNF-α) and interleukin-1 beta (IL-1β) [27].

These mechanisms are partly due to secondary metabolites evidenced by the metabolomic analysis, such as the alkaloid piplartine (**156**), a molecule involved in *Piper tuberculatum* activity and present in *P. longum* L. [28,29]. Several pharmacological activities have been attributed to this, specifically the gastroprotective effect. In this respect, piplartine (4.5 mg/kg of b.w.) reduced the acute lesions induced by EtOH, possibly by the maintenance of GSH levels and the basal gastric acid secretion in the gastric mucosa, and also by inhibiting the H^+^, K^+^-ATPase activity [28].

Other alkaloids evidenced in the present study were (E,E)-piperlonguminine (**37**), piperolactam D (**84**), and pipercitine (**161**).

Few metabolites present have antecedents to biological activities, which opens the opportunity to isolate them and evaluate whether they are responsible for the activity of *P. campylotropa* and elucidate other mechanisms of action.

## 4. Materials and Methods

### 4.1. Botanical and Chemical Characterization

#### 4.1.1. Plant Material

*Peperomia campylotropa* A.W. Hill was collected in February 2015 in the Municipality of Buenavista de Cuéllar, Guerrero, Mexico, and botanically authenticated by the Biologist María de la Luz Arreguín Sánchez, PhD (National School of Biological Sciences), with the help of the specialized literature [30] and compared to a voucher specimen deposited in the Herbarium of the National School of Biological Sciences, IPN (author Rzedowski, No. 29018).

The leaves and inflorescence (spikes) were dried entirely at room temperature, in the shade, and manually powdered with a mortar until they were of a medium particle size (approximately 1–2 mm). Then, they were stored in a hermetic container protected from light in a fresh (20–25 °C) and dry (60%) environment until used. The plant was used for no more than a year under these conditions.

#### 4.1.2. Ethnobotanical Study

The ethnobotanical study was conducted in the Municipality of Buenavista de Cuéllar, Guerrero, Mexico, using a previously identified specimen of *P. campylotropa*, shown to the people who voluntarily accepted to participate in the interview. After they indicated that they knew the species as a vendor, healer, or user, 10 questions were asked about the traditional use, the part consumed, their frequency of use, and the preparation of the species, among others.

Based on the analysis of these responses, we designed the study’s pharmacological conditions.

#### 4.1.3. Preparation of the Aqueous Extract of *P. campylotropa*

The aqueous extract was prepared daily as an infusion using the dry leaves and inflorescence (Section 4.1.1), which were weighed and placed in teabags to avoid losing particles in the final extract (250 mg/mL). The tea bag was introduced in a container with boiling water and left to stand for 5 min. From this extract, dilutions were carried out to obtain concentrations of 125 and 62.5 mg/mL, which were administered to rats in a volume of 1 mL/kg of body weight (b.w.) for final doses of 250, 125, and 62.5 mg/kg of b.w.

#### 4.1.4. Preliminary Phytochemical Screening

The extract was screened for secondary metabolites, such as alkaloids, flavonoids, cyanogenetic and cardiotonic glycosides, reducing sugars, saponins, tannins, quinones, coumarins, and sesquiterpene lactones, according to standard colorimetric and precipitation reactions proposed by Domínguez (1988) [13].

#### 4.1.5. Metabolomic Profile

The aqueous extract was chemically characterized under mass spectra using the Agilent Technologies 6545 QTOF LC/MS equipment (Agilent Technologies, UHPLC 1290 Infinity II, Santa Clara, CA, USA). The UHPLC analytical experiment of the extract was performed on a Zorbax Eclipse Plus C18 Column (2.1 × 50 mm; 1.8 µM). The separation of the components was performed by gradient elution from 0.1% aqueous formic acid (A) to 0.1% formic acid in acetonitrile (B) over 30 min (Table 4). The flow rate was 0.4 mL/min, and the injection volume was 2 µL. Milli-Q water was used as a blank. A sample of the aqueous extract was prepared at 10 mg/mL of Milli-Q water.

The metabolite database (METLIN database including Agilent QTOF mass spectrometers) identified individual secondary metabolites, which were ordered according to their increasing retention time (Table S1).

Score calculations were carried out through the signal with the highest intensity for each metabolite (main adduct), considering only clusters of adducts with more than one signal (no single ions function). Table S1 reports the score calculation from the main adduct.

#### 4.1.6. Gastroprotective Metabolites in the Aqueous Extract of *P. campylotropa*

Based on secondary metabolites identified in the metabolomic profile obtained by LC–MS (Table S1), we were interested in highlighting those previously reported for their gastroprotective applications and some related species in which these metabolites were associated.

The chemical classification and species related to the molecule were obtained by PubChem (https://pubchem.ncbi.nlm.nih.gov/, accessed on 26 July 2024). After that, a review of these in publications cataloged in Google Scholar using the keywords “antiulcerogenic”, “gastroprotective”, and “gastric ulcers” was performed (Table S2). Articles citing metabolites or other species related to them were considered to meet the inclusion criteria. Grouped results are shown in Section 2.3.

### 4.2. Pharmacological Evaluation

#### 4.2.1. Animals

Adult female Wistar rats (200 ± 20 g of b.w.) and NIH female mice (30 ± 5 g) were used for this study. They were housed and maintained in the animal house at room temperature (22–24 °C) and 50–55% relative humidity, with day/night cycles of 12 × 12 h. They were fed with a standard rodent diet and water ad libitum. Before the experiments, the rats fasted overnight with free access to water. Care and handling of the animals agreed with internationally accepted procedures following the recommendation indicated in the Mexican Technical Specifications for the Production, Care, and Use of Laboratory Animals [31] and approved by the Institutional Bioethics Committee (CEI-ENCB-009/2016).

#### 4.2.2. Indomethacin- and Absolute-Ethanol-Induced Gastric Damage

**Figure 6:**
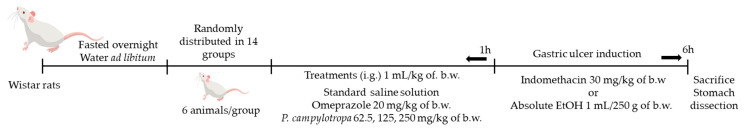
General scheme of experimental protocol. Self-authoring scheme. The rat image was designed by freepik.com (accessed on 31 January 2025). Premium license.

IND- and absolute-EtOH-induced gastric damage models were selected for *P. campylotropa* gastroprotective evaluation (Figure 6). According to Table 5, Wistar rats were randomized into 7 groups for each induction protocol. Gastric damage was induced according to the protocol established by Venkova et al. [32] with slight modifications by intragastric administration of an IND dose of 30 mg/kg of b.w. dissolved in 5% NaHCO_3_ or by an absolute-EtOH dose of 1 mL/250 g of b.w. [2].

In both gastric ulcer induction models, the treatments were administered by gavage in a volume of 1 mL/kg of b.w. by the intragastric route one hour before the ulcer induction. Later, animals were maintained in their home cages for 6 h; then, they were euthanized by cervical dislocation, and the stomachs were isolated to assess macroscopic damage. OMP (20 mg/kg of b.w.) was used as a reference drug in both models.

#### 4.2.3. Establishment of Endogenous NO, -SH Groups, and PG Role in the Gastroprotective Activity of *P. campylotropa*

**Figure 7:**
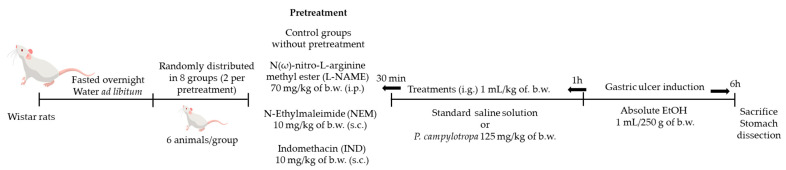
General scheme of experimental pretreatment protocol. Self-authoring scheme. The rat image was designed by freepik.com. Premium license.

To assess the involvement of endogenous inflammatory mediators in the gastroprotective effect of the aqueous extract of *P. campylotropa*, Wistar rats were randomly assigned into 8 groups (Table 6). N(ω)-nitro-L-arginine methyl ester (L-NAME), N-Ethylmaleimide (NEM), and IND were injected as pretreatments for inhibiting the NO, -SH groups, and PG, respectively, 30 min before the intragastric administration of NS or the aqueous extract at 125 mg/kg of b.w. After 1 h, absolute EtOH (1 mL/250 g of b.w.) was administered to each animal by the i.g. route. The animals were then maintained in their home cages for 2 h until they were euthanized by cervical dislocation. The stomachs were then isolated to assess macroscopic damage (Figure 7) [2,33].

#### 4.2.4. Assessment of Gastric Damage

An independent observer blinded during the treatment evaluated the severity of gastric damage. The stomach was dissected and opened along with the greater curvature. Samples were slightly cleaned with NS to remove food residues. Macroscopic damage was quantified by measuring the extent of the lesions in the stomach using photographs previously digitized and analyzed using the ImageJ program (imagej.nih.gov/ij/download/, accessed on 10 January, 2024).

The ulceration index (UI) was calculated for each sample by the following formulae:

UI = (damage total area/total stomach area) × 100

The gastroprotection percentage (%) was calculated according to the following:

% gastroprotection = (UI_C_ − UI_T_) × 100/UI_C_

UI_C_ is the ulceration index in the control groups (IND or EtOH), and UI_T_ is the ulceration index in the treatment groups.

#### 4.2.5. Acute Intragastric Toxicity in Mice

Acute intragastric toxicity was estimated in NIH female mice. Animals were distributed into five groups: Sham (I), and the aqueous extract of *P. campylotropa* (II–V) at 2.5, 5, 10, and 20 g/kg of b.w. The treatments were administered by gavage in a volume of 1 mL/kg of b.w. by the intragastric route. Animals were observed for seven days for any sign of toxicity.

#### 4.2.6. Statistical Analysis

All values are expressed as arithmetic means ± standard error (SEM). Data were calculated using Graph Pad Prism5^®^ Version 2.01 software. We used the Shapiro–Wilk test to test normal distribution. The statistical significance of each parameter was evaluated by a one-way analysis of variance (ANOVA) followed by Tukey’s test. *p*-values of < 0.05 were considered statistically significant.

## 5. Conclusions

This study demonstrated the gastroprotective activity of *P. campylotropa* A.W. Hill in a chemically induced gastric damage model in rats. This effect is possibly related to the 162 secondary metabolites characterized by UHPLC–MS, specifically those previously associated with gastroprotective activity: amarogentin, paeoniflorin, and piplartine.

The evidence also related to the participation of -SH groups in the gastroprotective mechanism of action of the aqueous extract.

These results contribute to understanding the chemical content of *P. campylotropa* and demonstrate its possible application as a gastroprotective agent; however, several studies are needed to deeply establish the mechanism of action and the components associated with this activity. Also, its chemical relationship with other species and families may provide evidence of other possible therapeutic applications.

## Figures and Tables

**Table 1:** Preliminary identification of secondary metabolites of *P. campylotropa* A.W. Hill.

Metabolite	Reaction
Flavonoids	
Flavones	Shinoda
Flavonols	10% Sodium hydroxide
Alkaloids	Dragendorff
Reducing sugars	Fehling
Tannins	1% Potassium ferricyanide
Saponins	
Triterpenoid	Rosenthaler

Metabolites were preliminary identified by protocols proposed by Domínguez (1988) [13].

**Table 2:** Percentage of gastroprotective effect of *P. campylotropa* extract on chemically induced ulceration models.

Treatment	Gastric Damage Inductor	Dose (mg/kg of b.w.)	Gastroprotection Percentage (%)
OMP	IND (30 mg/kg of b.w.)	20	73.90 ± 11.11
*P. campylotropa* aqueous extract		62.5	61.59 ± 15.22
		125	64.24 ± 15.89
		250	77.84 ± 12.20
OMP	EtOH (1 mL/250 g of b.w.)	20	80.81 ± 13.56
*P. campylotropa* aqueous extract		62.5	65.93 ± 12.71
		125	66.45 ± 13.15
		250	78.62 ± 20.80

Data are reported as means ± SEM obtained, as indicated in Section 4.2.4. IND = 30 mg/kg of b.w.; OMP = 20 mg/kg of b.w.; 62.5, 125, and 250 = groups treated with doses (mg/kg of b.w.) of 62.5, 125, and 250 of aqueous extract, respectively.

**Table 3:** Gastroprotective effect of *P. campylotropa* in rats administered with L-NAME, NEM, and IND subjected to EtOH-induced gastric lesions.

Group	UI	Gastroprotection Percentage (%)
NS-injected rats		
Sham	10.45 ± 1.79	-
*P. campylotropa*	1.20 ± 0.32 *	88.5
L-NAME-injected rats		
Sham	20.35 ± 3.38	-
*P. campylotropa*	8.75 ± 1.77 *	57.0
NEM-injected rats		
Sham	12.51 ± 2.16	-
*P. campylotropa*	17.33 ± 4.68 *	0.0
IND-injected rats		
Sham	11.25 ± 1.45	-
*P. campylotropa*	5.89 ± 0.88 *	47.6

Data are reported as means ± SEM obtained, as indicated in Section 4.2.4. n = 6. One-way analysis of variance and Tukey’s Multiple Comparison Test were carried out. * *p* < 0.05 = significantly different from their appropriate Sham group. NS = normal saline; IND = 10 mg/kg of b.w.; L-NAME = N(ω)-nitro-L-arginine methyl ester (70 mg/kg of b.w.); NEM = N-ethylmaleimide (10 mg/kg of b.w.); IND = indomethacin (10 mg/kg of b.w.); UI = ulceration index.

**Table 4:** Experimental parameters were used in the UHPLC–QTOF analysis to separate the metabolites from the aqueous extract of *P. campylotropa*.

Time (min)	Mobile Phase Composition	
	A %	B %
0.0	90.0	10.0
4.0	85.0	15.0
7.0	75.0	25.0
9.0	68.0	32.0
16.0	60.0	40.0
22.0	45.0	55.0
28.0	5.0	95.0
30.0	5.0	95.0

Note: UHPLC conditions: Zorbax Eclipse Plus 2.1X50 mm, 1.8 µm C18 Colum. Mobile phase A: 0.1% aqueous formic acid; mobile phase B: acetonitrile: 0.1% formic acid. Injection volume was 2 µL and flow rate of 0.4 mL/min. QTOF conditions: scanning mass from *m*/*z* 500 to 1000 and QTOF to m/z 50 to 1000 was operating in the positive-ion mode. Gas temperature, 280 °C; gas flow rate, 10 L/min; Nebulizer, 50 psi; cover gas temperature, 300 °C; cover gas flow, 10 L/min; VCap, 4000 V; Nozzle voltage, 500 V; fragmentor voltage, 110 V; Skimmer, 65 V; Octopolo RF Vpp, 750.

**Table 5:** Experimental groups for the induction of gastric damage.

Gastric Damage Model (Chemical Inductor)		No.	n	Group/Treatment (i.g.)
Indomethacin (IND)	Control groups	I	6	Sham Standard saline solution (NS)
		II	6	*P. campylotropa* Aqueous extract (250 mg/kg of b.w.)
	Damage groups (IND 30 mg/kg of b.w.)	III	6	IND Standard saline solution (NS)
		IV	6	OMP Omeprazole (20 mg/kg of b.w.)
		V	6	*P. campylotropa* Aqueous extract (62.5 mg/kg of b.w.)
		VI	6	*P. campylotropa* Aqueous extract (125 mg/kg of b.w.)
		VII	6	*P. campylotropa* Aqueous extract (250 mg/kg of b.w.)
Ethanol (EtOH)	Control groups	I	6	Sham Standard saline solution (NS)
		II	6	*P. campylotropa* Aqueous extract (250 mg/kg of b.w.)
	Damage groups (EtOH 1 mL/250 g of b.w.)	III	6	EtOH Standard saline solution (NS)
		IV	6	OMP Omeprazole (20 mg/kg of b.w.)
		V	6	*P. campylotropa* Aqueous extract (62.5 mg/kg of b.w.)
		VI	6	*P. campylotropa* Aqueous extract (125 mg/kg of b.w.)
		VII	6	*P. campylotropa* Aqueous extract (250 mg/kg of b.w.)

Wistar rats were randomly distributed in 14 groups, as shown in Table; n = number of animals per group. Treatments indicated in the last column were given intragastrically (i.g.) one hour before the ulcer induction.

**Table 6:** Pretreatment groups for establishing endogenous NO, -SH Groups, and PG roles in EtOH-induced gastric damage model.

Endogenous Molecule Involved	Pretreatment/Dose (Route)	No.	n	Group/Treatment (i.g.)
-	None	I	6	Sham Standard saline solution (NS)
		II	6	*P. campylotropa* Aqueous extract (125 mg/kg of b.w.)
Nitric oxide (NO)	N(ω)-nitro-L-arginine methyl ester (L-NAME) 70 mg/kg of b.w. (i.p.)	III	6	Sham Standard saline solution (NS)
		IV	6	*P. campylotropa* Aqueous extract (125 mg/kg of b.w.)
Sulfhydryl group (-SH)	N-Ethylmaleimide (NEM) 10 mg/kg of b.w. (s.c.)	V	6	Sham Standard saline solution (NS)
		VI	6	*P. campylotropa* Aqueous extract (125 mg/kg of b.w.)
Prostaglandins (PG)	Indomethacin (IND) 10 mg/kg of b.w. (s.c.)	VII	6	Sham Standard saline solution (NS)
		VIII	6	*P. campylotropa* Aqueous extract (125 mg/kg of b.w.)

Wistar rats were randomly distributed in 8 groups as shown in Table. n = number of animals per group. i.g.: intragastric; i.p.: intraperitoneal; s.c.: subcutaneous.

## Data Availability

Data are contained within the article or the Supplementary Materials.

## Supplementary Materials

The following supporting information can be downloaded at https://www.mdpi.com/article/10.3390/molecules30040772/s1, Figure S1: UHPLC–MS traces of the metabolomic profile of *P. campylotropa* A.W. Hill; Table S1: Secondary metabolites from the aqueous extract of *Peperomia campylotropa* obtained by the tandem mass metabolite database (METLIN database including Agilent QTOF mass spectrometers); Table S2: Type of secondary metabolites identified in *P. campylotropa* A.W. Hill by UHPLC–MS analysis and species in which they have been reported. References [18,28,29,34,35,36,37,38,39,40,41,42,43,44,45,46,47,48,49,50,51,52,53,54,55,56,57,58,59,60,61,62,63,64,65,66,67,68,69,70,71,72,73,74,75,76,77,78,79,80,81,82] are cited in Supplementary Materials.
