# Supplementary material for: Peperomia campylotropa A.W. Hill: Ethnobotanical, Phytochemical, and Metabolomic Profile Related to Its Gastroprotective Activity
Source: Molecules. 2025 Feb 7;30(4):772. doi: 10.3390/molecules30040772 (PMC11858570; doi:10.3390/molecules30040772)
Supplement: Supplementary file 1 [file molecules-30-00772-s001.zip › Figure S1.pdf]

Supplementary material

Figure S1. UHPLC-MS-traces of the metabolomic profile of *P. campylotropa* A.W. Hill

A

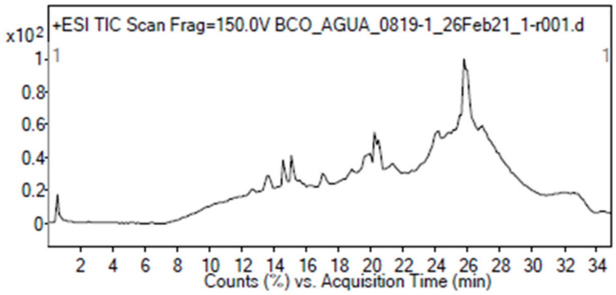

B

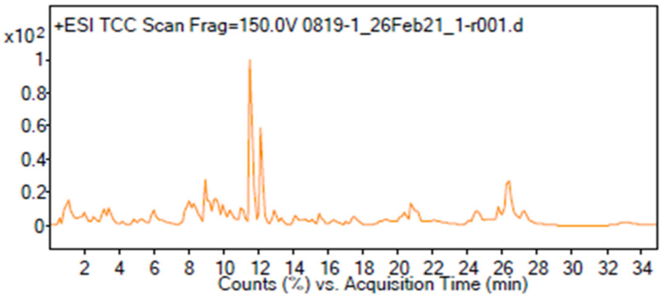

(A) Blanck traces and (B) Sample traces obtained by UHPLC-MS
