# Supplementary material for: Peperomia campylotropa A.W. Hill: Ethnobotanical, Phytochemical, and Metabolomic Profile Related to Its Gastroprotective Activity
Source: Molecules. 2025 Feb 7;30(4):772. doi: 10.3390/molecules30040772 (PMC11858570; doi:10.3390/molecules30040772)
Supplement: Supplementary file 1 [file molecules-30-00772-s001.zip › Table S1.pdf]

Supplementary material

Table S1. Secondary metabolites from the aqueous extract of *Peperomia campylotropa* obtained by tandem mass metabolite database (METLIN database including Agilent QTOF mass spectrometers).

ID	Compound	Retention Time (min)	QTOF analysis					
			Molecular Formula	Main Adduct	Exact Mass	Observed Mass	Score	Error (ppm)
**1**	(2S,2'R,3S,3'R,4S)-3,4',5,7-Tetrahydroxyflavan(2->7,4->8)-3,3',5,5',7-pentahydroxyflavan	0.827	C30 H24 O11	(M+H)+[-H2O]	560.1319	560.1332	73.00	-2.40
**2**	Tyramine glucuronide	0.971	C14 H19 N O7	(M+H)+[-H2O]	313.1162	313.1167	76.86	-1.64
**3**	Benzosimuline	0.973	C20 H19 N O2	(M+Na)+[-H2O]	305.1416	305.1401	76.31	4.75
**4**	Enicoflavine	0.977	C10 H13 N O4	(M+H)+[-H2O]	211.0845	211.0845	69.56	-0.16
**5**	Lasiocarpine	0.987	C21 H33 N O7	(M+K)+[-H2O]	411.2257	411.2244	51.84	3.25
**6**	6-O-Galloylsucrose	1.004	C19 H26 O15	(M+Na)+	494.1272	494.1293	67.16	-4.25
**7**	Vanilloloside	1.075	C14 H20 O8	(M+Na)+	316.1158	316.1161	60.85	-1.02
**8**	Tryptophol [xylosyl-(1->6)-glucoside]	1.087	C21 H29 N O10	(M+Na)+	455.1791	455.1803	70.92	-2.57
**9**	Ascorbigen	1.089	C15 H15 N O6	(M+H)+[-H2O]	305.0899	305.0891	80.57	2.58
**10**	Methyl 2-benzamidoacetate	1.093	C10 H11 N O3	(M+H)+[-H2O]	193.0739	193.0736	56.54	1.66
**11**	Lucuminamide	1.095	C19 H27 N O11	(M+Na)+[-H2O]	445.1584	445.1606	75.62	-4.86
**12**	Mescaline	1.096	C11 H17 N O3	(M+H)+[-H2O]	211.1208	211.1213	56.07	-2.21
**13**	Gentiobiosyl 2-methyl-6-oxo-2E,4E-heptadienoate	1.099	C20 H30 O13	(M+K)+	478.1686	478.1697	89.75	-2.13
**14**	Codeine-6-glucuronide	1.101	C24 H29 N O9	(M+H)+	475.1842	475.1833	55.56	1.92
**15**	Gravolenic acid	1.150	C14 H16 O6	(M+H)+[-H2O]	280.0947	280.0941	52.43	2.17
**16**	Demethylisoalangiside	1.159	C24 H29 N O10	(M+H)+	491.1791	491.1789	55.62	0.56
**17**	Citbrasine	1.171	C17H17NO6	(M+H)+[-H2O]	331.1056	331.1053	58.61	0.98
**18**	Medicarpin 3-O-glucoside-6'-malonate	1.182	C25 H26 O12	(M+H)+[-H2O]	518.1424	518.1415	62.85	1.78
**19**	cis-Zeatin riboside	1.220	C15 H21 N5 O5	(M+H)+[-H2O]	351.1543	351.1534	92.05	2.45
**20**	1,2,3,4-Tetrahydro-alpha,7-dihydroxy-beta-(hydroxymethyl)-9-methoxy-3,4-dioxocyclopenta[c][1]benzopyran-6-propanal	1.235	C17 H16 O8	(M+Na)+[-H2O]	348.0845	348.0853	82.28	-2.17
**21**	Picein	1.236	C14 H18 O7	(M+H)+[-H2O]	298.1053	298.1049	63.76	1.11
**22**	Avenanthramide G	1.340	C16 H13 N O5	(M+H)+	299.0794	299.0787	50.53	2.10
**23**	Semilepidinoside B	1.456	C17 H22 N2 O7	(M+H)+	366.1427	366.1426	81.66	0.33

**24**	Parsonsine	1.501	C22 H33 N O8	(M+Na)+[-H2O]	439.2206	439.221	63.96	-0.91
**25**	Demethyloleuropein	1.544	C24 H30 O13	(M+Na)+[-H2O]	526.1686	526.1668	58.96	3.53
**26**	Haplopine	1.577	C13 H11 N O4	(M+H)+	245.0688	245.0698	53.65	-3.94
**27**	Albomaculine	1.677	C19 H23 N O5	(M+H)+[-H2O]	345.1576	345.1591	93.40	-4.38
**28**	Acronycidine	1.747	C15 H15 N O5	(M+H)+	289.095	289.0955	84.27	-1.76
**29**	Linusitamarin	1.828	C17 H22 O9	(M+H)+[-H2O]	370.1264	370.1264	66.58	0.06
**30**	Tetrahydrocurcumin	1.831	C21 H24 O6	(M+Na)+[-H2O]	372.1573	372.1562	79.01	2.92
**31**	Candimine	1.875	C18 H19 N O6	(M+H)+[-H2O]	345.1212	345.1214	76.72	-0.41
**32**	Fulvine	1.995	C16 H23 N O5	(M+Na)+[-H2O]	309.1576	309.1588	95.56	-3.70
**33**	O-Methyllycorenine	1.998	C19 H25 N O4	(M+H)+[-H2O]	331.1784	331.1774	81.54	2.94
**34**	Balfourodine	2.119	C16 H19 N O4	(M+H)+[-H2O]	289.1314	289.1307	65.17	2.48
**35**	10-Deoxygeniposidic acid	2.152	C16 H22 O9	(M+Na)+[-H2O]	358.1264	358.1263	79.66	0.13
**36**	Sweroside	2.228	C16 H22 O9	(M+Na)+[-H2O]	358.1264	358.1242	59.30	6.00
**37**	(E,E)-Piperlonguminine	2.896	C16 H19 N O3	(M+H)+[-H2O]	273.1365	273.1369	75.99	-1.51
**38**	7-Hydroxy-3-(4-methoxyphenyl)-4-methylcoumarin	2.919	C17 H14 O4	(M+H)+[-H2O]	282.0892	282.0881	75.10	3.90
**39**	Hydroxysafflor yellow A	3.026	C27 H32 O16	(M+H)+[-H2O]	612.169	612.1678	72.55	2.03
**40**	Simmondsin 2'-ferulate	3.031	C26 H33 N O12	(M+H)+[-H2O]	551.2003	551.1992	75.87	1.98
**41**	7-Hydroxy-5-isopropyl-2-methoxy-3-methyl-1,4-naphthoquinone	3.053	C15 H16 O4	(M+Na)+[-H2O]	260.1049	260.1046	77.65	0.89
**42**	Citpressine I	3.462	C16 H15 N O5	(M+H)+	301.095	301.0955	83.23	-1.60
**43**	Caseadine	3.515	C20 H23 N O4	(M+H)+	341.1627	341.1638	93.12	-3.28
**44**	Daidzein 7-O-glucuronide	4.878	C21 H18 O10	(M+H)+	430.09	430.09	81.20	-0.05
**45**	Plantagoside	5.169	C21 H22 O12	(M+H)+[-H2O]	466.1111	466.1117	97.94	-1.34
**46**	Khellol glucoside	5.215	C19 H20 O10	(M+Na)+	408.1056	408.1066	78.31	-2.45
**47**	Mahaleboside	5.420	C15 H16 O8	(M+Na)+[-H2O]	324.0845	324.0842	80.46	0.91
**48**	Glucodistylin	5.469	C21 H22 O12	(M+H)+[-H2O]	466.1111	466.1104	97.46	1.54
**49**	3'-O-Methyl-(-)-epicatechin 7-O-glucuronide	5.999	C22 H24 O12	(M+H)+[-H2O]	480.1268	480.1277	96.48	-1.90
**50**	Formononetin 7-O-glucuronide	6.005	C22 H20 O10	(M+H)+[-H2O]	444.1056	444.1059	97.71	-0.60
**51**	1-Epideacetylbowdensine	6.013	C19 H23 N O6	(M+H)+[-H2O]	361.1525	361.1527	77.03	-0.36
**52**	3,5,8-Trihydroxy-3',4',7-trimethoxyflavone	6.045	C18 H16 O8	(M+H)+[-H2O]	360.0845	360.0849	99.28	-0.98
**53**	Diosmetin 7-O-beta-D-glucopyranoside	6.405	C22 H22 O11	(M+H)+[-H2O]	462.1162	462.1159	99.02	0.68
**54**	Betavulgarin xyloside	6.580	C22 H20 O10	(M+H)+[-H2O]	444.1056	444.1058	81.54	-0.43
**55**	Garcimangosone D	8.315	C19 H20 O9	(M+Na)+[-H2O]	392.1107	392.1089	72.24	4.77

**56**	3,4',5-Trihydroxy-3',7-dimethoxyflavanone	8.339	C17 H16 O7	(M+Na)+[-H2O]	332.0896	332.0884	74.54	3.58
**57**	(S)-Autumnaline	8.435	C21 H27 N O5	(M+H)+[-H2O]	373.1889	373.1879	94.95	2.78
**58**	(+)-Sophorol	8.643	C16 H12 O6	(M+H)+[-H2O]	300.0634	300.063	78.57	1.19
**59**	Allamandin	8.694	C15 H16 O7	(M+Na)+[-H2O]	308.0896	308.0882	90.08	4.39
**60**	5',8-Dihydroxy-3',4',7-trimethoxyflavan	8.889	C18 H20 O6	(M+Na)+	332.126	332.1261	69.29	-0.47
**61**	Dioscoretine	8.921	C13 H23 N O3	(M+Na)+[-H2O]	241.1678	241.1674	85.29	1.61
**62**	Erysodine	8.947	C18 H21 N O3	(M+H)+[-H2O]	299.1521	299.1522	69.35	-0.23
**63**	2'-Hydroxy-3,4',5',7,8-pentamethoxyflavone	8.955	C20 H20 O8	(M+Na)+[-H2O]	388.1158	388.1155	82.01	0.81
**64**	Apterin	8.955	C20 H24 O10	(M+Na)+[-H2O]	424.1369	424.1372	99.45	-0.56
**65**	Hesperetin 7-O-glucoside	8.955	C22 H24 O11	(M+H)+[-H2O]	464.1319	464.1304	94.56	3.13
**66**	Afzelechin 7-apioside	8.961	C20 H22 O9	(M+Na)+[-H2O]	406.1264	406.1264	99.27	-0.12
**67**	Muscomin	8.961	C18 H18 O7	(M+Na)+[-H2O]	346.1053	346.105	98.91	0.84
**68**	5,8-Dihydroxy-3-(4-hydroxybenzyl)-7-methoxy-4-chromanone 8-acetate	8.964	C19 H18 O7	(M+Na)+	358.1053	358.1051	83.79	0.55
**69**	trans-Grandmarin	8.972	C15 H16 O6	(M+Na)+[-H2O]	292.0947	292.0945	98.92	0.55
**70**	Pisatin	9.155	C17 H14 O6	(M+H)+[-H2O]	314.079	314.0796	82.78	-1.67
**71**	Physcionin	9.211	C22 H22 O10	(M+H)+[-H2O]	446.1213	446.1218	98.53	-1.19
**72**	Velloquercetin	9.212	C20 H16 O7	(M+H)+[-H2O]	368.0896	368.0885	95.82	2.98
**73**	Pinobanksin 3-O-acetate	9.218	C17 H14 O6	(M+H)+[-H2O]	314.079	314.0778	94.35	4.07
**74**	1-Caffeoyl-4-deoxyquinic acid	9.226	C16 H18 O8	(M+Na)+[-H2O]	338.1002	338.1006	72.96	-1.21
**75**	7-Hydroxy-4-methylphthalide O-[arabinosyl-(1->6)-glucoside]	9.291	C20 H26 O12	(M+Na)+[-H2O]	458.1424	458.1421	80.79	0.7
**76**	Multinoside A	9.294	C27 H30 O16	(M+H)+[-H2O]	610.1534	610.1507	88.79	4.37
**77**	Oxyayanin A	9.412	C18 H16 O8	(M+H)+[-H2O]	360.0845	360.0833	79.92	3.37
**78**	4',8-Dimethylgossypetin 3-glucoside	9.475	C23 H24 O13	(M+Na)+	508.1217	508.1222	75.52	-1.08
**79**	cis-p-Coumaric acid 4-[apiosyl-(1->2)-glucoside]	9.541	C20 H26 O12	(M+Na)+[-H2O]	458.1424	458.1433	78.59	-1.97
**80**	3,4,5-Trimethoxycinnamic acid	9.573	C12 H14 O5	(M+H)+[-H2O]	238.0841	238.0846	85.75	-2.18
**81**	4-Methoxycinnamic acid	9.588	C10 H10 O3	(M+H)+[-H2O]	178.063	178.0631	75.33	-0.36
**82**	3,5,6-Trihydroxy-3',4',7-trimethoxyflavone 3-glucuronide	9.612	C24 H24 O14	(M+H)+[-H2O]	536.1166	536.1157	87.62	1.77
**83**	1-O-E-Cinnamoyl-(6-arabinosylglucose)	9.615	C20 H26 O11	(M+Na)+[-H2O]	442.1475	442.1483	65.45	-1.72
**84**	Piperolactam D	9.625	C17 H13 N O4	(M+H)+[-H2O]	295.0845	295.0857	59.83	-4.06

**85**	4'-O-methyl-(-)-epicatechin-3'-O-beta-glucuronide	9.664	C23 H26 O12	(M+H)+[-H2O]	494.1424	494.1399	86.93	5.07
**86**	Phloridzin	9.667	C21 H24 O10	(M+Na)+	436.1369	436.1366	87.87	0.86
**87**	Byakangelicin	9.669	C17 H18 O7	(M+Na)+	334.1053	334.1044	94.79	2.68
**88**	(S)-Menthone 8-thioacetate	9.700	C12 H20 O2 S	(M+Na)+	228.1184	228.1191	66.24	-2.95
**89**	(+/-)-6-Acetonyldihydrochelerythrine	9.724	C24 H23 N O5	(M+H)+[-H2O]	405.1576	405.1596	73.62	-4.92
**90**	Dinorcapsaicin	9.773	C16 H23 N O3	(M+Na)+[-H2O]	277.1678	277.1687	66.41	-3.2
**91**	Hallactone B	9.804	C20 H24 O9 S	(M+H)+[-H2O]	440.1141	440.1147	57.93	-1.45
**92**	Methyl rosmarinate	9.837	C19 H18 O8	(M+H)+[-H2O]	374.1002	374.1007	83.09	-1.39
**93**	6''-O-Acetyldaidzin	9.838	C23 H22 O10	(M+H)+[-H2O]	458.1213	458.122	80.12	-1.54
**94**	beta-D-Gentiobiosyl crocetin	9.953	C32 H44 O14	(M+H)+[-H2O]	652.2731	652.2708	55.49	3.53
**95**	Biochanin A 7-O-(6-O-malonyl-β-D-glucoside)	9.961	C25 H24 O13	(M+H)+[-H2O]	532.1217	532.1215	97.73	0.27
**96**	6'-Malonyltrifolirhizin	9.963	C25 H24 O13	(M+H)+	532.1217	532.1225	96.58	-1.59
**97**	Erythratine	9.995	C18 H21 N O4	(M+H)+[-H2O]	315.1471	315.1469	79.71	0.53
**98**	Physagulin C	10.024	C30 H38 O9	(M+Na)+[-H2O]	542.2516	542.2537	64.97	-3.99
**99**	Kuwanon Z	10.050	C34 H26 O10	(M+H)+[-H2O]	594.1526	594.1551	81.89	-4.26
**100**	Isorhamnetin 3-O-[b-D-glucopyranosyl-(1->2)-a-L-rhamnopyranoside]	10.108	C28 H32 O16	(M+H)+[-H2O]	624.169	624.166	61.33	4.86
**101**	Caryatin glucoside	10.213	C23 H24 O12	(M+Na)+	492.1268	492.1267	98.80	0.16
**102**	3',8-Dimethoxyapigenin 7-glucoside	10.217	C23 H24 O12	(M+Na)+	492.1268	492.1262	98.75	1.22
**103**	Norhyoscyamine	10.277	C16 H21 N O3	(M+Na)+	275.1521	275.1512	60.99	3.53
**104**	Dracorubin	10.295	C32 H24 O5	(M+H)+	488.1624	488.1619	75.35	0.94
**105**	Cularidine	10.330	C19 H21 N O4	(M+H)+[-H2O]	327.1471	327.1463	76.72	2.45
**106**	Phenylethyl primeveroside	10.350	C19 H28 O10	(M+Na)+[-H2O]	416.1682	416.1691	81.56	-2.02
**107**	7-Ethoxy-4-methyl-2H-1-benzopyran-2-one	10.361	C12 H12 O3	(M+H)+[-H2O]	204.0786	204.0788	87.14	-0.62
**108**	Pancracine	10.366	C16 H17 N O4	(M+H)+[-H2O]	287.1158	287.1165	56.16	-2.46
**109**	Cymorcin diglucoside	10.378	C22 H34 O12	(M+H)+[-H2O]	490.205	490.2038	75.39	2.45
**110**	Ankorine	10.413	C19 H29 N O4	(M+H)+	335.2097	335.2098	84.64	-0.51
**111**	Quercetin 3-[rhamnosyl-(1->2)-alpha-L-arabinopyranoside]	10.440	C26 H28 O15	(M+H)+[-H2O]	580.1428	580.1434	97.99	-1.06
**112**	Maclurin 3-C-(6''-p-hydroxybenzoyl-glucoside)	10.449	C26 H24 O13	(M+H)+	544.1217	544.1222	73.55	-0.87
**113**	Thamnosin	10.545	C30 H28 O6	(M+Na)+[-H2O]	484.1886	484.1875	76.24	2.31
**114**	Sciadopitysin	10.601	C33 H24 O10	(M+H)+[-H2O]	580.1369	580.1394	81.84	-4.15
**115**	Salviaflaside	10.608	C24 H26 O13	(M+Na)+	522.1373	522.1361	75.32	2.33

**116**	7-Methyl-1,4,5-naphthalenetriol 4-[xylosyl-(1->6)-glucoside]	10.785	C22 H28 O12	(M+Na)+[-H2O]	484.1581	484.1565	73.08	3.33
**117**	cis-Zeatin 9-glucoside	10.839	C16 H23 N5 O6	(M+Na)+	381.1648	381.1646	79.55	0.56
**118**	Gigantine	11.002	C13 H19 N O3	(M+Na)+[-H2O]	237.1365	237.1373	83.95	-3.35
**119**	Salviaflaside methyl ester	11.129	C25 H28 O13	(M+H)+[-H2O]	536.153	536.1533	94.24	-0.67
**120**	1-Peroxyferolide	11.329	C17 H22 O7	(2M+H)+	338.1366	338.1357	65.81	2.56
**121**	Crinamidine	11.380	C17 H19 N O5	(M+H)+	317.1263	317.1254	73.59	2.8
**122**	Thalsimine	11.497	C38 H40 N2 O7	(M+Na)+	636.2836	636.2813	89.16	3.55
**123**	Cnidimol 7-glucoside	11.510	C21 H26 O10	(M+Na)+[-H2O]	438.1526	438.1524	99.43	0.51
**124**	2,3-Dihydro-2-(4-hydroxyphenyl)-5,6,7,8-tetramethoxy-4H-1-benzopyran-4-one	11.511	C19 H20 O7	(M+Na)+[-H2O]	360.1209	360.1199	97.12	2.69
**125**	Piceid	11.511	C20 H22 O8	(M+Na)+[-H2O]	390.1315	390.1303	90.69	2.99
**126**	4',7-Di-O-methylcatechin	11.512	C17 H18 O6	(M+Na)+	318.1103	318.1096	97.71	2.4
**127**	b-D-fructosyl-a-D-(6-O-(E))-feruloylglucoside	11.630	C21 H28 O12	(M+Na)+[-H2O]	472.1581	472.1579	75.88	0.4
**128**	7-Hydroxy-3,4',8-trimethoxyflavone	11.759	C18 H16 O6	(M+H)+[-H2O]	328.0947	328.0934	78.31	3.98
**129**	Nobiletin	11.759	C21 H22 O8	(M+Na)+[-H2O]	402.1315	402.1326	80.32	-2.69
**130**	Wistin	11.759	C23 H24 O10	(M+H)+[-H2O]	460.1369	460.1363	97.39	1.43
**131**	(Z)-Resveratrol 4'-glucoside	11.761	C20 H22 O8	(M+Na)+[-H2O]	390.1315	390.1324	76.45	-2.49
**132**	Austrobailignan	11.762	C21 H18 O7	(M+H)+[-H2O]	382.1053	382.1043	96.97	2.55
**133**	8-Hydroxy-4',5,7-trimethoxyflavone	11.767	C18 H16 O6	(M+H)+[-H2O]	328.0947	328.0946	99.61	0.34
**134**	Norrubrofusarin 6-beta-gentiobioside	11.832	C26 H30 O15	(M+Na)+	582.1585	582.1597	72.45	-2.03
**135**	10-Acetoxyoleuropein	12.077	C27 H34 O15	(M+Na)+	598.1898	598.1907	69.46	-1.53
**136**	Biochanin A 7-(6-methylmalonylglucoside)	12.144	C26 H26 O13	(M+H)+[-H2O]	546.1373	546.1371	98.41	0.42
**137**	Gardenin B	12.145	C19 H18 O7	(M+H)+[-H2O]	358.1053	358.1043	81.70	2.68
**138**	Apigenin 6-C-glucoside 8-C-arabinoside	12.146	C26 H28 O14	(M+H)+[-H2O]	564.1479	564.1472	95.11	1.17
**139**	3-(5,6,6-Trimethylbicyclo[2.2.1]hept-1-yl)cyclohexanol	12.489	C16 H28 O	(M+K)+[-H2O]	236.214	236.2144	69.05	-1.65
**140**	3,4,5-Trimethoxyphenyl 2,6-digalloylglucoside	12.651	C29 H30 O17	(M+H)+[-H2O]	650.1483	650.1483	74.48	-0.06
**141**	Amarogentin	12.798	C29 H30 O13	(M+H)+	586.1686	586.1678	71.20	1.51
**142**	Sterebin A	12.813	C18 H30 O4	(M+H)+[-H2O]	310.2144	310.2141	69.92	1.00
**143**	Chryso-obtusin glucoside	12.907	C25 H28 O12	(M+H)+[-H2O]	520.1581	520.1572	97.38	1.70
**144**	Paeonilactone C	12.909	C17 H18 O6	(M+Na)+	318.1103	318.111	82.87	-2.24
**145**	Isoquercitrin 4''-rhamnoside	12.945	C27 H30 O16	(M+Na)+	610.1534	610.1541	72.12	-1.11

**146**	Napelline	13.037	C22 H33 N O3	(M+Na)+[-H2O]	359.246	359.2451	80.75	2.67
**147**	ent-16-Kauren-19-ol acetate	13.076	C22 H34 O2	(M+Na)+[-H2O]	330.2559	330.2539	53.73	6.02
**148**	Paeoniflorin	13.116	C23 H28 O11	(M+Na)+[-H2O]	480.1632	480.1636	80.40	-0.98
**149**	Bufotenine O-glucoside	13.125	C18 H26 N2 O6	(M+H)+	366.1791	366.178	77.79	3.02
**150**	Flavonol 3-O-D-glycoside	13.166	C21 H20 O8	(M+H)+[-H2O]	400.1158	400.1155	82.36	0.84
**151**	Silidianin	13.166	C25 H24 O10	(M+H)+	484.1369	484.1364	76.20	1.17
**152**	6''-Acetylapiin	13.304	C28 H30 O15	(M+H)+[-H2O]	606.1585	606.1591	97.24	-0.97
**153**	Epilubimin	13.308	C15 H24 O2	(M+H)+[-H2O]	236.1776	236.1782	86.02	-2.24
**154**	Ferulic acid	14.713	C10 H10 O4	(M+H)+[-H2O]	194.0579	194.0588	83.02	-4.40
**155**	(S)-Annocherine A	15.013	C17 H15 N O4	(M+H)+[-H2O]	297.1001	297.1002	66.12	-0.41
**156**	Piplartine	15.371	C17 H19 N O5	(M+Na)+[-H2O]	317.1263	317.1268	83.20	-1.59
**157**	3-Methyl-alpha-ionyl acetate	16.549	C16 H26 O2	(M+H)+	250.1933	250.1943	82.53	-4.04
**158**	3-(3,4-Dihydroxybenzyl)-7-hydroxy-5-methoxy-4-chromanone	17.092	C17 H16 O6	(M+H)+[-H2O]	316.0947	316.0959	93.79	-3.73
**159**	Sagequinone methide A	18.528	C20 H24 O4	(M+H)+[-H2O]	328.1675	328.168	67.60	-1.51
**160**	Sterebin D	18.869	C18 H30 O3	(M+H)+[-H2O]	294.2195	294.2195	85.42	-0.15
**161**	Pipercitine	19.046	C23 H43 N O	(M+H)+[-H2O]	349.3345	349.335	84.19	-1.60
**162**	(1S,3R,4R)-8,10-Dihydroxyfenchone 10-O-b-D-glucoside	20.881	C16 H26 O8	(M+H)+[-H2O]	346.1628	346.1629	99.50	-0.52

Secondary metabolites were characterized as indicated in Section 4.1.5. Metabolomic Profile. Metabolites are order by increasing retention time criteria.
