# Supplementary material for: Peperomia campylotropa A.W. Hill: Ethnobotanical, Phytochemical, and Metabolomic Profile Related to Its Gastroprotective Activity
Source: Molecules. 2025 Feb 7;30(4):772. doi: 10.3390/molecules30040772 (PMC11858570; doi:10.3390/molecules30040772)
Supplement: Supplementary file 1 [file molecules-30-00772-s001.zip › Table S2.pdf]

Supplementary material

Table S2. Type of secondary metabolites identified in *P. campylotropa* A.W. Hill by UHPLC-MS analysis and species in which they have been reported.

ID	Compound	Type of secondary metabolite*	Plant species related to* (Family)
**1**	(2S,2'R,3S,3'R,4S)-3,4',5,7-Tetrahydroxyflavan(2->7,4->8)-3,3',5,5',7-pentahydroxyflavan	flavonoid oligomer	---
**2**	Tyramine glucuronide	glycoside	---
**3**	Benzosimuline	alkaloid	*Zanthoxylum simulans* (Rutaceae)
**4**	Enicoflavine	delta-lactone	*Swertia ciliata* (Gentianaceae)
**5**	Lasiocarpine	alkaloid	*Heliotropium ellipticum* (Boraginaceae) *Heliotropium arbainense* (Boraginaceae)
**6**	6-O-Galloylsucrose	tannin	*Paeonia obovata* (Paeoniaceae) [34] *Rheum palmatum* (Polygonaceae) *Rheum coreanum* (Polygonaceae)
**7**	Vanilloloside	glycoside	*Hypericum erectum* (Hypericaceae) *Itoa orientalis* (Salicaceae)
**8**	Tryptophol [xylosyl-(1->6)-glucoside]	glycoside	---
**9**	Ascorbigen	phenolic glycoside	*Arabidopsis thaliana* (Brasicaceae)
**10**	Methyl 2-benzamidoacetate	glycine derivative	*Brassica napus* (Brasicaceae)
**11**	Lucuminamide	glycoside	
**12**	Mescaline	phenethylamine alkaloid	*Senegalia berlandieri* (Fabaceae) *Gymnocalycium chubutense* (Cactaceae)
**13**	Gentiobiosyl 2-methyl-6-oxo-2E,4E-heptadienoate	glycoside	---
**14**	Codeine-6-glucuronide	morphinane alkaloid	---
**15**	Gravolenic acid	hydroxycinnamic acid	---
**16**	Demethylisoalangiside	glycoside	*Carapichea ipecacuana* (Rubiaceae)
**17**	Citbrasine	alkaloid	*Swinglea glutinosa* (Rutaceae)

**18**	Medicarpin 3-O-glucoside-6'-malonate	pterocarpan	*Maackia amurensis* (Fabaceae)
**19**	cis-Zeatin riboside	glycoside	*Medicago sativa* (Fabaceae) [35]<br>*Ipomoea batatas* (Convolvulaceae) [36–38]
**20**	1,2,3,4-Tetrahydro-alpha,7-dihydroxy-beta-(hydroxymethyl)-9-methoxy-3,4-dioxocyclopenta[c][1]benzopyran-6-propanal	coumarin	---
**21**	Picein	phenolic compound	*Rhodiola crenulate* (Crassulaceae)<br>*Salvia officinalis* (Lamiaceae) [39,40]
**22**	Avenanthramide G	phenolic compound	---
**23**	Semilepidinoside B	phenolic glycoside	*Lepidium sativum* (Brassicaceae)
**24**	Parsonsine	alkaloid	*Parsonsia alboflavescens* (Apocynaceae)
**25**	Demethyloleuropein	phenolic compound	*Jasminum grandiflorum* (Oleaceae) [41,42]<br>*Olea europaea* (Oleaceae) [43]
**26**	Haplopine	alkaloid	*Zanthoxylum simulans* (Rutaceae)<br>*Melicope semecarpifolia* (Rutaceae)
**27**	Albomaculine	alkaloid	*Hosta plantaginea* (Asparagaceae)
**28**	Acronycidine	quinoline alkaloid	*Sarcomelicope argyrophylla* (Rutaceae)<br>*Medicosma fareana* (Rutaceae)
**29**	Linusitamarin	glycoside	*Linum usitatissimum* (Linaceae) [44]
**30**	Tetrahydrocurcumin	curcumin	*Curcuma longa* (Zingiberaceae) [45–49]
**31**	Candimine	alkaloid	*Hippeastrum morelianum* (Amaryllidaceae)
**32**	Fulvine	pyrrolizidine alkaloid	*Crotalaria crispata* (Fabaceae)<br>*Crotalaria madurensis* (Fabaceae)
**33**	O-Methyllycorenine	alkaloid	*Hymenocallis littoralis* (Amaryllidaceae)<br>*Hosta plantaginea* (Asparagaceae)

**34**	Balfourodine	alkaloid	*Balfourodendron riedelianum* (Rutaceae)
**35**	10-Deoxygeniposidic acid	terpen glycoside	*Scyphiphora hydrophyllacea* (Rubiaceae)
**36**	Sweroside	glycoside	*Gentiana macrophylla* (Gentianaceae)<br>*Gentiana algida* (Gentianaceae)
**37**	(E,E)-Piperlonguminine	amide alkaloid	*Piper khasianum* (Piperaceae)<br>*Piper attenuatum* (Piperaceae) [18]
**38**	7-Hydroxy-3-(4-methoxyphenyl)-4-methylcoumarin	coumarin	---
**39**	Hydroxysafflor yellow A	phenolic compound	*Carthamus tinctorius* (Asteraceae) [50,51]
**40**	Simmondsin 2'-ferulate	cyanoglycoside	*Simmondsia chinensis* (Simmondsiaceae)
**41**	7-Hydroxy-5-isopropyl-2-methoxy-3-methyl-1,4-naphthoquinone	naphthoquinone	*Pachira aquatica* (Bombacaceae)
**42**	Citpressine I	alkaloid	*Citrus sulcata* (Rutaceae)<br>*Citrus reticulata* (Rutaceae) [52]
**43**	Caseadine	alkaloid	*Dasymaschalon sootepense* (Annonaceae)<br>*Corydalis caseana* (Papaveraceae)<br>*Ceratocapnos heterocarpa* (Papaveraceae)
**44**	Daidzein 7-O-glucuronide	isoflavonoid	---
**45**	Plantagoside	tetrahydroxyflavanone	*Plantago depressa* (Plantaginaceae)<br>*Plantago asiatica* (Plantaginaceae)
**46**	Khellol glucoside	glycoside	*Ammi visnaga* (Apiaceae) [53]
**47**	Mahaleboside	coumarin	---
**48**	Glucodistylin	flavonoid	*Rhododendron flammeum* (Ericaceae)<br>*Hibiscus mutabilis* (Mavaceae)
**49**	3'-O-Methyl-(-)-epicatechin 7-O-glucuronide	flavonoid	---
**50**	Formononetin 7-O-glucuronide	isoflavonoid	---
**51**	1-Epideacetylbowdensine	alkaloid	*Crinum moorei* (Amaryllidaceae)

			Brunsvigia radulosa (Amaryllidaceae)
**52**	3,5,8-Trihydroxy-3',4',7-trimethoxyflavone	hydroxyflavan	---
**53**	Diosmetin 7-O-beta-D-glucopyranoside	flavone	*Ferulopsis hystrix* (Apiaceae) [54]<br>*Mentha spicata* (Lamiaceae)
**54**	Betavulgarin xyloside	isoflavone	---
**55**	Garcimangosone D	glycoside	*Hypericum sampsonii* (Hypericaceae)<br>*Polygala karensium* (Polygalaceae)
**56**	3,4',5-Trihydroxy-3',7-dimethoxyflavanone	flavonoid	*Pulicaria canariensis* (Asteraceae)<br>*Artemisia crithmifolia* (Asteraceae)
**57**	(S)-Autumnaline	isoquinoline alkaloid	*Colchicum autumnale* (Colchicaceae)<br>*Colchicum ritchii* (Colchicaceae)
**58**	(+)-Sophorol	isoflavanone	*Dalbergia parviflora* (Fabaceae)
**59**	Allamandin	terpen lactone	*Plumeria rubra* (Apocynaceae)<br>*Allamanda schottii* (Apocynaceae)
**60**	5',8-Dihydroxy-3',4',7-trimethoxyflavan	flavonoid	---
**61**	Dioscoretine	alkaloid	*Dioscorea dumetorum* (Dioscoreaceae)
**62**	Erysodine	alkaloid	*Erythrina stricta* (Fabaceae)<br>*Erythrina leptorhiza* (Fabaceae)
**63**	2'-Hydroxy-3,4',5',7,8-pentamethoxyflavone	flavone	*Parkia biglobosa* (Fabaceae)<br>*Mimosa diplotricha* (Fabaceae)<br>*Mimosa invisa* (Fabaceae)
**64**	Apterin	furanocoumarin	*Peucedanum japonicum* (Apiaceae)<br>*Heracleum yungningense* (Apiaceae)
**65**	Hesperetin 7-O-glucoside	flavanone	*Cunila* (Lamiaceae)
**66**	Afzelechin 7-apioside	hydroxyflavan	---
**67**	Muscomin	homoflavonoid	---
**68**	5,8-Dihydroxy-3-(4-hydroxybenzyl)-7-methoxy-4-chromanone 8-acetate	homoflavonoid	---
**69**	trans-Grandmarin	coumarin	*Citrus tamurana* (Rutaceae)

**70**	Pisatin	pterocarpan	*Tephrosia candida* (Leguminosae)<br>*Lathyrus sativus* (Leguminosae)
**71**	Physcionin	anthraquinone	*Rheum palmatum* (Polygonaceae)
**72**	Velloquercetin	flavonoid	*Cunila* (Lamiaceae)<br>*Vellozia* (Velloziaceae)
**73**	Pinobanksin 3-O-acetate	dihydroflavonol	*Populus yunnanensis* (Salicaceae)
**74**	1-Caffeoyl-4-deoxyquinic acid	hydroxycinnamic acid	---
**75**	7-Hydroxy-4-methylphthalide O-[arabinosyl-(1->6)-glucoside]	glycoside	---
**76**	Multinoside A	glycosyloxyflavone	*Sinocrassula indica* (Crassulaceae)<br>*Rosa multiflora* (Rosaceae)<br>*Prunus tomentosa* (Rosaceae)
**77**	Oxyayanin A	trihydroxyflavone	*Apuleia leiocarpa* (Leguminosae)<br>*Distemonanthus benthamianus* (Fabaceae)<br>*Waltheria indica* (Malvaceae)
**78**	4',8-Dimethylgossypetin 3-glucoside	flavonol	---
**79**	cis-p-Coumaric acid 4-[apiosyl-(1->2)-glucoside]	glycoside	---
**80**	3,4,5-Trimethoxycinnamic acid	coumaric acid	*Polygala tenuifolia* (Polygalaceae)<br>*Piper swartzianum* (Piperaceae)<br>*Piper tuberculatum* (Piperaceae) [28]
**81**	4-Methoxycinnamic acid	phenolic acid	*Baccharis subdentata* (Asteraceae)<br>*Scrophularia buergeriana* (Scrophulariaceae)
**82**	3,5,6-Trihydroxy-3',4',7-trimethoxyflavone 3-glucuronide	flavonoid	---
**83**	1-O-E-Cinnamoyl-(6-arabinosylglucose)	glycoside	---
**84**	Piperolactam D	alkaloid	*Piper arborescens* (Piperaceae)<br>*Piper attenuatum* (Piperaceae) [18]
**85**	4'-O-methyl-(-)-epicatechin-3'-O-beta-glucuronide	flavonoid	---
**86**	Phloridzin	dihydrochalcone	*Lithocarpus pachyphyllus* (Fagaceae)<br>*Malus doumeri* (Rosaceae)
**87**	Byakangelicin	furanocoumarin	*Angelica japonica* (Apiaceae)

			Heracleum grandiflorum (Apiaceae)
**88**	(S)-Menthone 8-thioacetate	p-menthane monoterpenoid	---
**89**	(+/-)-6-Acetonyldihydrochelerythrine	benzophenanthridine alkaloid	*Chelidonium majus* (Papaveraceae) [55]<br>*Zanthoxylum nitidum* (Rutaceae) [56]
**90**	Dinorcapsaicin	phenolic compound	*Capsicum annuum* (Solanaceae) [57]
**91**	Hallactone B	diterpenoid	*Podocarpus hallii* (Podocarpaceae)<br>*Podocarpus macrophyllus* (Podocarpaceae)
**92**	Methyl rosmarinate	hydroxycinnamic acid	*Salvia miltiorrhiza* (Lamiaceae)<br>*Perilla frutescens* (Lamiaceae) [58]
**93**	6''-O-Acetyldaidzin	isoflavonoid	*Glycine max* (Fabaceae) [59,60]<br>*Hibiscus syriacus* (Malvaceae)
**94**	beta-D-Gentiobiosyl crocetin	glycoside	*Gardenia jasminoides* (Rubiaceae) [61]<br>*Crocus sativus* (Iridaceae) [62]
**95**	Biochanin A 7-O-(6-O-malonyl-β-D-glucoside)	isoflavone	---
**96**	6'-Malonyltrifolirhizin	pterocarpan	*Maackia amurensis* (Fabaceae)<br>*Cicer judaicum* (Fabaceae)<br>*Sophora flavescens* (Fabaceae)
**97**	Erythratine	alkaloid	*Erythrina fusca* (Fabaceae)<br>*Erythrina herbacea* (Fabaceae)
**98**	Physagulin C	withanolide	---
**99**	Kuwanon Z	flavan	---
**100**	Isorhamnetin 3-O-[b-D-glucopyranosyl-(1->2)-a-L-rhamnopyranoside]	flavonoid	*Ginkgo biloba* (Ginkgoaceae) [63]
**101**	Caryatin glucoside	flavonoid	---
**102**	3',8-Dimethoxyapigenin 7-glucoside	flavonoid	---
**103**	Norhyoscyamine	alkaloid	*Cyphanthera odgersii* (Solanaceae)<br>*Cyphanthera tasmanica* (Solanaceae)
**104**	Dracorubin	proanthocyanidin	*Calamus draco* (Arecaceae)

**105**	Cularidine	alkaloid	*Ceratocapnos claviculate* (Papaveraceae)<br>*Sarcocapnos crassifolia* (Papaveraceae)
**106**	Phenylethyl primeveroside	glycoside	*Camellia sinensis* (Theaceae) [64–67]<br>*Rehmannia glutinosa* (Scrophulariaceae)<br>*Sida cordifolia* (Malvaceae)
**107**	7-Ethoxy-4-methyl-2H-1-benzopyran-2-one	coumarin	---
**108**	Pancracine	isoquinoline alkaloid	*Pancratium maritimum* (Amaryllidaceae)<br>*Amaryllis belladonna* (Amaryllidaceae)
**109**	Cymorcin diglucoside	glycoside	---
**110**	Ankorine	benzo[a]quinolizidine alkaloid	*Alangium lamarckii* (Cornaceae)<br>*Alangium salviifolium* (Cornaceae)
**111**	Quercetin 3-[rhamnosyl-(1->2)-alpha-L-arabinopyranoside]	flavonoid	*Brassica nigra* (Brassicaceae)
**112**	Maclurin 3-C-(6''-p-hydroxybenzoyl-glucoside)	glycoside	*Mangifera indica* (Anacardiaceae) [68–71]
**113**	Thamnosin	coumarin	*Thamnosma montana* (Rutaceae)<br>*Citrus maxima* (Rutaceae)
**114**	Sciadopitysin	hydroxyflavone	*Taxus cuspidate* (Taxaceae)<br>*Torreya nucifera* (Cephalotaxaceae)
**115**	Salviaflaside	glycoside	*Perilla frutescens* (Lamiaceae) [58]<br>*Salvia deserti* (Lamiaceae)
**116**	7-Methyl-1,4,5-naphthalenetriol 4-[xylosyl-(1->6)-glucoside]	glycoside	---
**117**	cis-Zeatin 9-glucoside	glycoside	*Solanum lycopersicum* (Solanaceae)
**118**	Gigantine	isoquinoline	*Carnegiea gigantea* (Cactaceae)
**119**	Salviaflaside methyl ester	glycoside	---
**120**	1-Peroxyferolide	gamma-lactone	*Liriodendron tulipifera* (Magnoliaceae)
**121**	Crinamidine	alkaloid	*Crinum moorei* (Amaryllidaceae)

			Brunsvigia orientalis (Amaryllidaceae)
**122**	Thalsimine	isoquinoline	*Thalictrum simplex* (Ranunculaceae)
**123**	Cnidimol 7-glucoside	glycoside	---
**124**	2,3-Dihydro-2-(4-hydroxyphenyl)-5,6,7,8-tetramethoxy-4H-1-benzopyran-4-one	methoxy flavanone	---
**125**	Piceid	glycoside	*Humulus lupulus* (Cannabaceae) *Veratrum dahuricum* (Melanthiaceae)
**126**	4',7-Di-O-methylcatechin	catechin	*Solanum lycopersicum* (Solanaceae)
**127**	b-D-fructosyl-a-D-(6-O-(E))-feruloylglucoside	cinnamate ester	---
**128**	7-Hydroxy-3,4',8-trimethoxyflavone	flavonoid	*Parkia biglobosa* (Fabaceae)
**129**	Nobiletin	hexamethoxyflavone	---
**130**	Wistin	isoflavonoid	*Bowdichia virgilioides* (Fabaceae) *Glycyrrhiza pallidiflora* (Fabaceae)
**131**	(Z)-Resveratrol 4'-glucoside	glycoside	---
**132**	Austrobailignan 1	lactone	*Austrobaileya scandens* (Austrobaileyaceae) *Amyris pinnata* (Rutaceae)
**133**	8-Hydroxy-4',5,7-trimethoxyflavone	flavonoid	*Citrus reticulata* (Rutaceae) [52] *Citrus deliciosa* (Rutaceae)
**134**	Norrubrofusarin 6-beta-gentiobioside	glycoside	---
**135**	10-Acetoxyoleuropein	terpene glycoside	*Osmanthus fragrans* (Oleaceae) *Osmanthus heterophyllus* (Oleaceae) *Olea europaea* (Oleaceae) [43]
**136**	Biochanin A 7-(6-methylmalonylglucoside)	isoflavonoid	---
**137**	Gardenin B	tetramethoxyflavone	*Citrus tankan* (Rutaceae) *Citrus reticulata* (Rutaceae) [52]
**138**	Apigenin 6-C-glucoside 8-C-arabinoside	flavonoid	*Nelumbo nucifera* (Nelumbonaceae) [72]

**139**	3-(5,6,6-Trimethylbicyclo[2.2.1]hept-1-yl)cyclohexanol	monoterpenoid	---
**140**	3,4,5-Trimethoxyphenyl 2,6-digalloylglucoside	glycoside	*Lotus japonicus* (Fabaceae)
**141**	Amarogentin [73,74]	secoiridoid glycoside	*Swertia japónica* (Gentianaceae) [73] *Gentianella nítida* (Gentianaceae)
**142**	Sterebin A	sesquiterpenoid	*Stevia rebaudiana* (Asteraceae) [75]
**143**	Chryso-obtusin glucoside	anthraquinone	*Senna obtusifolia* (Fabaceae) *Senna tora* (Fabaceae)
**144**	Paeonilactone C	monoterpenic lactone	*Paeonia lactiflora* (Paeoniaceae) [34]
**145**	Isoquercitrin 4''-rhamnoside	flavonoid	---
**146**	Napelline	kaurane diterpenoid	*Aconitum baicalense* (Ranunculaceae) *Aconitum volubile* (Ranunculaceae)
**147**	ent-16-Kauren-19-ol acetate	kaurane diterpenoid	---
**148**	Paeoniflorin [34,76]	terpene glycoside	*Paeonia emodi* (Paeoniaceae) *Paeonia obovata* (Paeoniaceae) [34]
**149**	Bufotenine O-glucoside	glycoside	---
**150**	Flavonol 3-O-D-glycoside	glycosiloxyflavone	---
**151**	Silidianin	flavanone	*Silybum eburneum* (Asteraceae) *Silybum marianum* (Asteraceae) [77,78]
**152**	6''-Acetylapiin	flavonoid	*Petroselinum crispum* (Apiaceae) [79] *Linum suffruticosum* (Linaceae)
**153**	Epilubimin	sesquiterpenoid	*Hyoscyamus albus* (Solanaceae) [80] *Solanum aethiopicum* (Solanaceae) *Solanum melongena* (Solanaceae) [81]
**154**	Ferulic acid [82]	polyphenolic compound	*Salvia rosmarinus* (Lamiaceae) *Camellia reticulata* (Theaceae)
**155**	(S)-Annocherine A	isoquinoline	---
**156**	Piplartine [28,29]	amide alkaloid	*Piper arborescens* (Piperaceae)

			Piper puberulum (Piperaceae)
**157**	3-Methyl-alpha-ionyl acetate	sesquiterpenoid	*Arabidopsis thaliana* (Brassicaceae)
**158**	3-(3,4-Dihydroxybenzyl)-7-hydroxy-5-methoxy-4-chromanone	homoflavonoid	*Muscari* (Asparagaceae)
**159**	Sagequinone methide A	diterpene lactone	*Salvia officinalis* (Lamiaceae) [39,40]
**160**	Sterebin D	sesquiterpenoid	---
**161**	Pipercitine	alkaloid	*Piper nigrum* (Piperaceae)
**162**	(1S,3R,4R)-8,10-Dihydroxyfenchone 10-O-b-D-glucoside	terpene glycoside	---

Types of metabolites and species-related were obtained as indicated in Section 4.1.6. Gastroprotective metabolites in the aqueous extract of P. *campylotropa*.

18 Soni, G.K.; Sharma, S.; Dangi, N. In Silico Molecular Docking Study and Protective Effect of Piper Attenuatum on Aspirin Induced Gastric Ulcer in Rats. *Curr. Chem. Lett.* **2023**, *12*, 705–720. https://doi.org/10.5267/j.ccl.2023.5.004.

28 Burci, L.M.; Pereira, I.T.; Da Silva, L.M.; Rodrigues, R.V.; Facundo, V.A.; Militão, J.S.L.T.; Santos, A.R.S.; Marques, M.C.A.; Baggio, C.H.; Werner, M.F.D.P. Antiulcer and Gastric Antisecretory Effects of Dichloromethane Fraction and Piplartine Obtained from Fruits of *Piper tuberculatum* Jacq. in Rats. *J. Ethnopharmacol.* **2013**, *148*, 165–174. https://doi.org/10.1016/j.jep.2013.04.006.

29 Do Nascimento, R.F.; De Sales, I.R.P.; De Oliveira Formiga, R.; Barbosa-Filho, J.M.; Sobral, M.V.; Tavares, J.F.; De Fátima Formiga Melo Diniz, M.; Batista, L.M. Activity of Alkaloids on Peptic Ulcer: What's New? *Molecules* **2015**, *20*, 929–950.

34. Bae, J.Y.; Kim, C.Y.; Kim, H.J.; Park, J.H.; Ahn, M.J. Differences in the Chemical Profiles and Biological Activities of Paeonia Lactiflora and Paeonia Obovata. *J. Med. Food* **2015**, *18*, 224–232. https://doi.org/10.1089/jmf.2014.3144.
35. Chandra, P.; Kaleem, M.; Sachan, N.; Pathak, R.; Alanazi, A.S.; Alsaif, N.A.; Alsanea, S.; Alsuwayt, B.; Alanazi, M.M.; Kabra, A. Gastroprotective Evaluation of *Medicago sativa* L. (Fabaceae) on Diabetic Rats. *Saudi Med. J.* **2023**, *31*, 101815. https://doi.org/10.1016/j.jsps.2023.101815.
36. Panda, V.; Sonkamble, M.; Sanjeev Panda, V.; Kundnani, P.K.M. Anti-Ulcer Activity of Ipomoea Batatas Tubers (Sweet Potato). *Funct. Food Health Dis.* **2012**, *2*, 48–61.
37. Hermes, D.; Dudek, D.N.; Maria, M.D.; Horta, L.P.; Lima, E.N.; De Fátima, Â.; Sanches, A.C.C.; Modolo, L.V. In Vivo Wound Healing and Antiulcer Properties of White Sweet Potato (Ipomoea Batatas). *J. Adv. Res.* **2013**, *4*, 411–415. https://doi.org/10.1016/j.jare.2012.06.001.
38. Jabar, H.; Almuoswi, N.; Al-Bideri, A.W.M. Histological and Immunohistochemical Study of the Healing Role of the Extract of Ipomoea Batatas Sweet Potato Extract in the Gastric Ulcer Developed by Aspirin in Male Albino Rats. *Eurasia J. Biosci.* **2019**, *13*, 399–407.
39. Mayer, B.; Baggio, C.H.; Freitas, C.S.; dos Santos, A.C.; Twardowschy, A.; Horst, H.; Pizzolatti, M.G.; Micke, G.A.; Heller, M.; dos Santos, É.P.; et al. Gastroprotective Constituents of *Salvia officinalis* L. *Fitoterapia* **2009**, *80*, 421–426. https://doi.org/10.1016/j.fitote.2009.05.015.
40. Fiorentin, T.R.; De Mello, M.B.; Aquino, A.M.K.; Rigo, B.A.; Loss, C.G.; Schwanz, M.; Junior, A.E.H.; Macedo, S.M.D. Antiulcerogenic Potential of *Salvia officinalis* L. Extract in Rats. *J. Appl. Pharm. Sci.* **2013**, *3*, 32–35. https://doi.org/10.7324/JAPS.2013.3806.

41. Umamaheswari, M.; Asokkumar, K.; Rathidevi, R.; Sivashanmugam, A.T.; Subhadradevi, V.; Ravi, T.K. Antiulcer and in Vitro Antioxidant Activities of *Jasminum grandiflorum* L. *J. Ethnopharmacol.* **2007**, *110*, 464–470. https://doi.org/10.1016/j.jep.2006.10.017.
42. Hunasagi, B.S.; Dhole, S.N. Antiulcer Potential of Ethanolic Extract of *Jasminum grandiflorum* Linn. Leaves on Different Ulcer Models. *World J. Pharm. Res.* **2022**, *11*, 2343–2359. https://doi.org/10.20959/wjpr20222-23121.
43. Al-Quraishy, S.; Othman, M.S.; Dkhil, M.A.; Abdel Moneim, A.E. Olive (Olea Europaea) Leaf Methanolic Extract Prevents HCl/Ethanol-Induced Gastritis in Rats by Attenuating Inflammation and Augmenting Antioxidant Enzyme Activities. *Biomed. Pharmacother.* **2017**, *91*, 338–349. https://doi.org/10.1016/j.biopha.2017.04.069.
44. Ibrahim, H.; Telep, N.; Hemed, M. Moringa (Moringa Oleifera) Leaves' Extract and Linseed (Linum Usitatissimum) Oil Ameliorate Piroxicam Induced Gastric Ulcers in Male Rats. *Zagazig Vet. J.* **2019**, *47*, 419–431. https://doi.org/10.21608/zvjz.2019.14491.1057.
45. Liju, V.B.; Jeena, K.; Kuttan, R. Gastroprotective Activity of Essential Oils from Turmeric and Ginger. *J. Basic. Clin. Physiol. Pharmacol.* **2015**, *26*, 95–103. https://doi.org/10.1515/jbcpp-2013-0165.
46. Yadav, S.; Sah, A.K.; Jha, R.; Sah, P.; Shah, D. Turmeric (Curcumin) Remedies Gastroprotective Action. *Pharmacogn. Rev.* **2013**, *7*, 42–46. https://doi.org/10.4103/0973-7847.112843.
47. Ikhueoya Airaodion, A.; Oluwaseun Obajimi, O.; Nancy Ezebuiro, C.; Ogbuagu, U.; Paul Agunbiade, A.; Paul Oloruntoba, A.; Dada Akinmolayan, J.; Rebecca Adeniji, A.; Oloseuan Airaodion, E. Prophylactic Efficacy of Aqueous Extract of Curcuma Longa against Indomethacin-Induced Ulcer in Male Wistar Rats. *Int. J. Res.* **2019**, *6*, 87–91.
48. Kim, D.-C.; Kim, S.-H.; Choi, B.-H.; Baek, N.-I.; Kim, D.; Kim, M.-J.; Kim, K.-T. Curcuma Longa Extract Protects against Gastric Ulcers by Blocking H2 Histamine Receptors. *Biol. Pharm. Bull.* **2005**, *28*, 2220–2224.
49. Rafatullah, S.; Tariq, M.; Al-Yahya, M.A.; Mossa, J.S.; Ageel, A.M. Evaluation of Turmeric (Curcuma Longa) for Gastric and Duodenal Antiulcer Activity in Rats. *J. Ethnopharmacol.* **1990**, *29*, 25–34.
50. Mandade, R.; Sreenivas, S.A.; Sakarkar, D.M.; Wanare, R. Pharmacological Effects of Extract of Carthamus Tinctorius on Volume and Acidity of Stimulated Gastric Secretion. *Orient. Pharm. Exp. Med.* **2011**, *11*, 293–298. https://doi.org/10.1007/s13596-011-0041-3.
51. Gupta, A.; Sheth, N.R.; Pandey, S.; Yadav, J.S.; Shah, D.R.; Vyas, B.; Joshi, S. Antiulcer Screening of Carthamus Tinctorius on Volume and Acidity of Stimulated Gastric Secretion in Rats. *J. Pharmacol. Pharmacother.* **2012**, *3*, 183–185. https://doi.org/10.4103/0976-500X.95523.
52. Li, Q.; Lu, T.; Chen, X.; Chu, H. Composition and Anti-Ulcer Activity of the Essential Oil from Citri Reticulatae Pericarpium in Rodents. *Braz. Arch. Biol. Technol.* **2023**, *66*, e23210786. https://doi.org/10.1590/1678-4324-2023210786.
53. Saleh, A. Gastroprotective and Antioxidative Effects of Ammi Visnaga Extract Against Ethanol-Induced Gastric Mucosal Ulceration in Male Albino Rats. *Iran. J. Toxicol.* **2023**, *17*, 137–146.
54. Razuvaeva, Y.G.; Toropova, A.A.; Salchak, S.M.; Olennikov, D.N. Coumarins of Ferulopsis Hystrix: LC–MS Profiling and Gastroprotective and Antioxidant Activities of Skimmin and Peucenidin. *Appl. Sci.* **2023**, *13*, 9653. https://doi.org/10.3390/app13179653.
55. Maji, A.K.; Banerji, P. *Chelidonium majus* L. (Greater Celandine)—A Review on Its Phytochemical and Therapeutic Perspectives. *Int. J. Herb. Med.* **2015**, *3*, 10–27.
56. Siddiqui, N.A.; Nooreen, Z.; Wal, P.; Yadav, A.K.; I Fantoukh, O.; Alqasoumi, S.I.; Ahmad, A.; Nasir, A. Evaluation of Antiulcer Potential of Tambulin and Ombuin Isolated from Zanthoxylum Armatum. *J. King Saud. Univ. Sci.* **2024**, *36*, 103326. https://doi.org/10.1016/j.jksus.2024.103326.
57. Srinivas, T.L.; Lakshmi, S.M.; Shama, S.N.; Reddy, G.K.; Prasanna, K.R. Medicinal Plants as Anti-Ulcer Agents. *J. Pharmacogn. Phytochem.* **2013**, *2*, 91–97.
58. Kangwan, N.; Pintha, K.; Lekawanvijit, S.; Suttajit, M. Rosmarinic Acid Enriched Fraction from Perilla Frutescens Leaves Strongly Protects Indomethacin-Induced Gastric Ulcer in Rats. *BioMed Res. Int.* **2019**, *1*, 9514703. https://doi.org/10.1155/2019/9514703.

59. Olugbesan, A.; Salami, A.T.; Odukanmi, A.O.; Omayone, T.; Iyiola, T.O.; Olaleye, S.B. Effects of Diets Containing Seeds of Soybean (Glycine Max) on Healing of Acetic Acid Induced Gastric Ulcers in Rats. *Arch. Basic. Appl. Med.* **2018**, *6*, 159–165.
60. Sowndhararajan, K.; Paul, S.; Kwon, G.S.; Hwang, C.W.; Kang, S.C. Protective Effect of Polyamine Extract of Salt Stressed and Sprouted Soybean Seeds against Ethanol-Induced Gastric Ulcer in Rats. *Food Sci. Biotechnol.* **2014**, *23*, 711–716. https://doi.org/10.1007/s10068-014-0096-2.
61. Lee, Y.I.; Hussain, A.; Rahman, A.A.; Sohn, H.Y.; Yoon, H.J.; Cho, J.S. Preventive Effect of LS-RUG-Com-a Mixture of Rubus Crataegifolius, Ulmus Macrocarpa, and Gardenia Jasminoides-on Gastric Disorders in Animal Models. *J. Life Sci.* **2023**, *33*, 923–935. https://doi.org/10.5352/JLS.2023.33.11.923.
62. Al-Mofleh, I.A.; Alhaider, A.A.; Mossa, J.S.; Al-Sohaibani, M.O.; Qureshi, S.; Rafatullah, S. Antigastric Ulcer Studies on "saffron" *Crocus sativus* L. in Rats. *PBMJ* **2006**, *9*, 1009–1013. https://doi.org/10.3923/pjbs.2006.1009.1013.
63. El-Tanbouly, G.S.; Mahmoud, M.F.; Mohamed, M.A. Gastroprotective Effect of Standardized Ginkgo Bioloba Extract (EGB761) against Indomethacin-Induced Gastric Ulcers in Rats. *DUSJ* **2020**, *3*, 8.
64. Rodrigues, S.F.M.; Ribeiro da Silva, L.M.; Gomes, D.A.S.; da Silva, M.C.E.; Campos, A.R.; Rodrigues, H.D.K.; Silva de Lima, A.C.; Bruno de Sousa, S.L.; Wilane de Figueiredo, R. Microencapsulation of Green Tea Extract (*Camellia sinensis* Var Assamica) to Increase the Bioaccessibility of Bioactive Compounds and Gastroprotective Effects. *Food Biosci.* **2021**, *42*, 101190. https://doi.org/10.1016/j.fbio.2021.101190.
65. Scoparo, C.T.; Borato, D.G.; Souza, L.M.; Dartora, N.; Silva, L.M.; Maria-Ferreira, D.; Sassaki, G.L.; Gorin, P.A.J.; Baggio, C.H.; Iacomini, M. Gastroprotective Bio-Guiding Fractionation of Hydro-Alcoholic Extracts from Green- and Black-Teas (*Camellia sinensis*). *Food Res. Int.* **2014**, *64*, 577–586. https://doi.org/10.1016/j.foodres.2014.07.043.
66. Ratnasooriya, W.; Fernando, T. Gastric Ulcer Healing Activity of Sri Lankan Black Tea (*Camellia sinensis* L.) in Rats. *Pharmacogn. Mag.* **2009**, *4*, 260–265.
67. Borato, D.G.; Scoparo, C.T.; Maria-Ferreira, D.; Da Silva, L.M.; De Souza, L.M.; Iacomini, M.; Werner, M.F.D.P.; Baggio, C.H. Healing Mechanisms of the Hydroalcoholic Extract and Ethyl Acetate Fraction of Green Tea (*Camellia sinensis* (L.) Kuntze) on Chronic Gastric Ulcers. *Naunyn Schmiedebergs Arch. Pharmacol.* **2016**, *389*, 259–268. https://doi.org/10.1007/s00210-015-1200-8.
68. Ferreira, G.C.C.; de Siqueira, O.L.; Rodrigues, D.C.; Ribeiro, P.R.V.; Canuto, K.M.; Duarte, A.S.G.; Eça, K.S.; de Figueiredo, R.W. Evidence for Antioxidant and Anti-Inflammatory Potential of Mango (*Mangifera indica* L.) in Naproxen-Induced Gastric Lesions in Rat. *J. Food Biochem.* **2022**, *46*, e13880. https://doi.org/10.1111/jfbc.13880.
69. Khan, N.; Khushtar, M.; Rahman, M.A.; Kaish, M.; Ajmal, M. Amelioration of Gastric Ulcer Using a Hydro-Alcoholic Extract of *Mangifera indica* in Sprague Dawley Rats by Prevention of Muco-Oxidative Stress. *Pharmacol. Res. Mod. Chin. Med.* **2024**, *11*, 100442. https://doi.org/10.1016/j.prmcm.2024.100442.
70. Severi, J.A.; Lima, Z.P.; Kushima, H.; Brito, A.R.M.S.; Santos, L.C. Dos; Vilegas, W.; Hiruma-Lima, C.A. Polyphenols with Antiulcerogenic Action from Aqueous Decoction of Mango Leaves (*Mangifera indica* L.). *Molecules* **2009**, *14*, 1098–1110. https://doi.org/10.3390/molecules14031098.
71. Sahu, P.K.; Martha, S.K.; Pradhan, D. Gastro-Protective Activity of Leaves of *Mangifera indica* Un Ulcer Induced Albino Rats. *World J. Pharm. Res.* **2017**, *6*, 685. https://doi.org/10.20959/wjpr201717-10339.
72. Srivastava, A.K.; Kumar Patil, U.; Singhai, A.; Kumar, M. Anti-Ulcer and Antioxidant Activity of Nelumbo Nucifera Gaertn Stalks in Rats. *Int. J. Pharm. Pharm. Sci.* **2015**, *7*, 3.
73. Niiho, Y.; Yamazaki, T.; Nakajima, Y.; Yamamoto, T.; Ando, H.; Hirai, Y.; Toriizuka, K.; Ida, Y. Gastroprotective Effects of Bitter Principles Isolated from Gentian Root and Swertia Herb on Experimentally-Induced Gastric Lesions in Rats. *J. Nat. Med.* **2006**, *60*, 82–88. https://doi.org/10.1007/s11418-005-0014-2.
74. Selvaperumal, M.K.N.S.; Arumugam, G.; Saranya, P. Gastroprotective Effect of Swertia Chirayita—A Study with Ulcer Induced Rats. *Pharmacologyonline* **2010**, *1*, 332–355.
75. Pandiyan, R.; Kannan, V.R. Effect on Extract of Stevia Rebaudiana Bertoni. In Ethanol Induced Gastric Ulcer by Using Wister Rats. *Recent. Res. Sci. Technol.* **2009**, *1*, 127130.

76. Wang, S.; Ni, Y.; Liu, J.; Yu, H.; Guo, B.; Liu, E.; He, J.; Wang, X.; Zhang, Y.; Wang, T. Protective Effects of Weilikang Decoction on Gastric Ulcers and Possible Mechanisms. *J. Nat. Med.* **2016**, *70*, 391–403. https://doi.org/10.1007/s11418-016-0985-1.
77. Alarcón De Lastra, C.; Martin, M.; Motilva, V.; Jiménez, M.; La Casa, C.; López, A. Gastroprotection Induced by Silymarin, the Hepatoprotective Principle of Silybum Marianum in Ischemia-Reperfusion Mucosal Injury: Role of Neutrophils. *Planta Med.* **1995**, *61*, 116–119.
78. Khayyal, M.T.; El-Ghazaly, M.A.; Kenawy, S.A.; Seif-El-Nasr, M.; Mahran, L.G.; Kafafi, Y.A.H.; Okpanyi, S.N. Antiulcerogenic Effect of Some Acting Plant Extracts and Their Combination. *Arzneim.-Forsch./Drug Res.* **2001**, *51*, 545–553.
79. Al-Howiriny, T.; Al-Sohaibani, M.; El-Tahir, K.; Rafatullah, S. Prevention of Experimentally-Induced Gastric Ulcers in Rats by an Ethanolic Extract of "Parsley" Petroselinum Crispum. *Am. J. Chin. Med.* **2003**, *31*, 699–711.
80. Yahia, M.; Yahia, M.; Benhouda, A.; Benbia, S.; Khadraoui, H. Ulcer Healing and Gastroprotective Activity of Methanolic Extracts of Hyoscyamus Albus and Umbilicus Rupestris Leaves against Gastric Injury Caused by Ethanol in Rats. *Glob. J. Med. Res.* **2017**, *4*, 8. https://doi.org/10.21767/2393-8854.10008.
81. El-Feky, A.M.; Aboul Naser, A.F.; Hamed, M. Solanum Melongena Peels against Ethanol-Induced Gastric Ulcer in Rats via Regulating Mucosal Enzymes, Oxidative Stress and Inflammatory Mediators´pathways. *Egypt. J. Chem.* **2024**, *67*, 587–600. https://doi.org/10.21608/ejchem.2024.271316.9354.
82. Ermis, A.; Aritici Colak, G.; Acikel-Elmas, M.; Arbak, S.; Kolgazi, M. Ferulic Acid Treats Gastric Ulcer via Suppressing Oxidative Stress and Inflammation. *Life* **2023**, *13*, 388. https://doi.org/10.3390/life13020388.
